# Single-cell analysis of murine fibroblasts identifies neonatal to adult switching that regulates cardiomyocyte maturation

**DOI:** 10.1038/s41467-020-16204-w

**Published:** 2020-05-22

**Authors:** Yin Wang, Fang Yao, Lipeng Wang, Zheng Li, Zongna Ren, Dandan Li, Mingzhi Zhang, Leng Han, Shi-qiang Wang, Bingying Zhou, Li Wang

**Affiliations:** 1 State Key Laboratory of Cardiovascular Disease, Fuwai Hospital, National Center for Cardiovascular Diseases, Chinese Academy of Medical Sciences and Peking Union Medical College, Beijing, 100037 China; 2 State Key Laboratory of Membrane Biology, College of Life Sciences, Peking University, Beijing, 100871 China; 3 Department of Biochemistry and Molecular Biology, The University of Texas Health Science Center at Houston McGovern Medical School, Houston, TX 77030 USA

**Keywords:** Developmental biology, Cardiovascular biology

## Abstract

Cardiac maturation lays the foundation for postnatal heart development and disease, yet little is known about the contributions of the microenvironment to cardiomyocyte maturation. By integrating single-cell RNA-sequencing data of mouse hearts at multiple postnatal stages, we construct cellular interactomes and regulatory signaling networks. Here we report switching of fibroblast subtypes from a neonatal to adult state and this drives cardiomyocyte maturation. Molecular and functional maturation of neonatal mouse cardiomyocytes and human embryonic stem cell-derived cardiomyocytes are considerably enhanced upon co-culture with corresponding adult cardiac fibroblasts. Further, single-cell analysis of in vivo and in vitro cardiomyocyte maturation trajectories identify highly conserved signaling pathways, pharmacological targeting of which substantially delays cardiomyocyte maturation in postnatal hearts, and markedly enhances cardiomyocyte proliferation and improves cardiac function in infarcted hearts. Together, we identify cardiac fibroblasts as a key constituent in the microenvironment promoting cardiomyocyte maturation, providing insights into how the manipulation of cardiomyocyte maturity may impact on disease development and regeneration.

## Abstract (web summary)

How cardiomyocytes mature and what regulates this is unclear. Here, the authors use single-cell analysis to examine how the population of murine cardiac fibroblasts changes during development and affects maturation of cardiomyocytes.

## Introduction

Unlike other cell types in the heart, cardiomyocytes (CMs) are terminally differentiated cells that lack proliferative capacity to repair myocardial damage caused by disease or other insults. Therefore, restoring the loss of cardiomyocytes remains a fundamental challenge in the treatment of cardiac injury, such as myocardial infarction. In past decades, enormous efforts were undertaken to reconstitute damaged cardiac tissues, typically using exogenous cell-based or cell-free therapies, which revolutionized our understanding of cardiac regeneration^1–9^. However, many hurdles still lie ahead on their road into the clinic. Adverse effects, such as arrhythmia and teratoma formation, were frequently observed in studies using pluripotent stem cell-derived cardiomyocytes due to their immature nature^1,4^. Another strategy commonly attempted was to arouse intrinsic programs to force endogenous mature cardiomyocytes back into the cell cycle, so as to supply the damaged myocardium via proliferation, whose reparative effects were marginal nonetheless^2,3,10–14^. Hence, development of more promising therapies requires an update on our understanding of cardiomyocyte maturation.

Compared to well-established mechanisms of cardiac fate commitment in embryonic development^15–21^, postnatal cardiomyocyte maturation is much less well molecularly defined, which many recent studies have started to address^22,23^. A panoply of factors, including transcription factors, microRNAs, as well as endothelial nitric oxide synthase (eNOS), were suggested to play crucial roles in the maturation of the conduction system in the heart, or in controlling other aspects of cardiomyocyte biology, such as metabolism, cell size, contractility, and proliferation^20,24,25^. These and other studies together suggested multi-layered regulatory networks in cardiomyocyte maturation, urging for more detailed and comprehensive characterization of the underlying molecular events.

The cell microenvironment is known to play critical roles in regulating cell fate by producing biophysical and biochemical cues, yet little is known about its roles in cardiomyocyte maturation. A recent study reported enhanced maturation of human embryonic stem cell-derived cardiomyocytes in a 3D tissue-engineered culture environment^26^. Alternatively, advanced cardiac maturation can be achieved by physical conditioning with increasing intensity over time, further underscoring the importance of the microenvironment in cardiac fate decisions^27^. In contrast to the biophysical environment, biochemical influences on cardiomyocyte maturation remain to be explored in greater depth.

In fact, postnatal heart development offers natural clues to understanding alterations in the cellular microenvironment, as well as their impact on the maturation of cardiomyocytes. Here, we apply single-cell RNA sequencing to analyze the cellular composition and interactions at different postnatal developmental stages, and unveil cardiac fibroblasts as the major constituent in the cellular niche driving cardiomyocyte maturation. Importantly, manipulation of fibroblasts or downstream signaling pathways regulates cardiomyocyte maturity, providing potentially viable strategies to improve outcomes of stem cell-based or cell-free therapies.

## Results

### Single-cell analysis of murine postnatal heart development

**Fig. 1:**
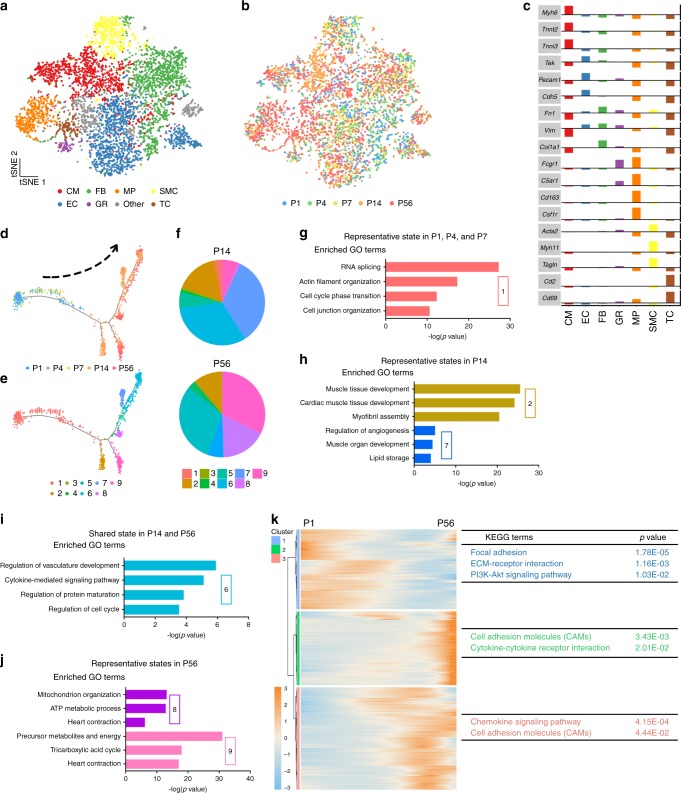
Single-cell analysis of murine postnatal heart development. **a**, **b** *t*-distributed stochastic neighbor embedding (*t*-SNE) clustering of single cells from postnatal day 1 (P1), P4, P7, P14, and P56 hearts. CCA was applied to remove batch effects. Cells were colored by cell type (**a**) or time point (**b**). CM cardiomyocyte, EC endothelial cell, FB fibroblast, MP macrophage, SMC smooth muscle cell, TC T cell, GR granulocyte. **c** Cell populations in **a** were identified by the expression of established marker genes. **d**, **e** Monocle analyses showing the ordering of CMs in pseudotime. Each color indicates either a time point (**d**) or a cell state (**e**). **f** State proportions of CMs from P14 or P56 hearts, respectively. **g**–**j** Gene ontology (GO) analysis of genes specifically expressed in a given CM state from **e**. Selected top categories are shown here (Supplementary Data 1–6). **k** Heatmap to display different blocks of top 1000 differentially expressed genes (DEGs) along the pseudotime trajectory (**d**, **e**). Please see Supplementary Data 7 for the full list. Right: selected top Kyoto Encyclopedia of Genes and Genomes (KEGG) terms related to corresponding DEGs in blocks significantly changed during cardiomyocyte maturation (P1–P56), Functional analysis was performed with enrichKEGG in clusterProfiler, *p* < 0.05 was considered significant enrichment.

To understand the molecular underpinnings of cardiomyocyte maturation, we set out to investigate single-cell transcriptomic profiles of mouse cardiac cells at various postnatal developmental stages. To this end, we first acquired a single-cell RNA-Sequencing (scRNA-Seq) dataset of left ventricles from mouse hearts at postnatal days (P) 1, 4, 7, and 14 from a separate study. To further capture the mature cardiac state, we performed scRNA-Seq on left ventricles of P56-hearts using the same experimental design and platform (Supplementary Fig. 1a). The image-based selection of individual cells used in our system assured the reliability of sample input, precluding dead cells and multiplets (Supplementary Fig. 1a). A total of 2497 cells, including both cardiomyocytes (CMs) and non-cardiomyocytes (NCMs), from two P56-mouse hearts, were sequenced (Supplementary Fig. 1b), of which 2137 qualified for subsequent analysis upon stringent filtering (please see “Methods” for details regarding quality control). All data (P1, P4, P7, P14, and P56) reached a median depth of 233,320 reads/cell, 84% alignment rate/cell, and 2610 genes/cell (Supplementary Fig. 1c–e). Following quality control, we performed *t*-distributed stochastic neighbor embedding (*t*-SNE) analysis with Seurat to define major cell clusters in P56 hearts, which included cardiomyocyte (CM), endothelial cell (EC), fibroblast (FB), macrophage (MP), smooth muscle cell (SMC), T cell (TC), and granulocyte (GR) clusters, based on their respective molecular signatures (Fig. 1a–c). Possible batch/individual effects caused by single-cell chips or individual animals were ruled out by indistinguishable cell clustering using 107 housekeeping genes (Supplementary Fig. 1f), as well as close clustering of cells of a given cell type across different time points (Fig. 1a, b). Next, to depict progressively changing molecular features of CMs during maturation, we ordered all CMs in pseudotime to reconstruct the trajectory of cardiomyocyte maturation (Fig. 1d, e). While CMs from P1, P4, and P7 mainly contributed to State 1, P14-CMs, and P56-CMs were segregated into multiple states in pseudotime. Specifically, State 2 and 7 were mainly composed of CMs from P14, whereas States 8 and 9 were dominated by P56-CMs. State 6, however, emerged as a unique state with balanced contributions from both P14- and P56-CMs (Fig. 1d–f). Gene ontology (GO) analysis showed that genes highly expressed in State 1 (representative of P1-CMs, P4-CMs, and P7-CMs) were enriched in functions related to RNA splicing, cell cycle phase transition, etc. (Fig. 1g). Genes highly expressed in States 2 and 7 (representative of P14-CMs) were enriched in functions related to cardiac muscle development, whereas those specifically expressed in States 8 and 9 (representative of P56-CMs) were associated with heart contraction and ATP metabolic process (Fig. 1h–j). Further, genes abundantly expressed in State 6 (shared state of P14-CMs and P56-CMs) were implicated in the regulation of vasculature development, protein maturation, and cell cycle (Fig. 1i). These functional enrichments closely reflected molecular features of CMs from immature to mature states. Moving forward, Kyoto Encyclopedia of Genes and Genomes (KEGG) Pathway analysis of differentially expressed genes (DEGs) along the P1–P56 maturation route identified multiple signaling pathways implicated in maturation, including chemokine signaling pathways, cytokine–cytokine receptor interactions, ECM–receptor interactions, PI3K-Akt signaling, etc (Fig. 1k). Together, these findings demonstrated the transcriptional complexity of cardiomyocyte maturation, and suggested important pathways that could be exploited for regulatory purposes.

### Prediction of cardiac fibroblasts as pro-maturation factor

**Fig. 2:**
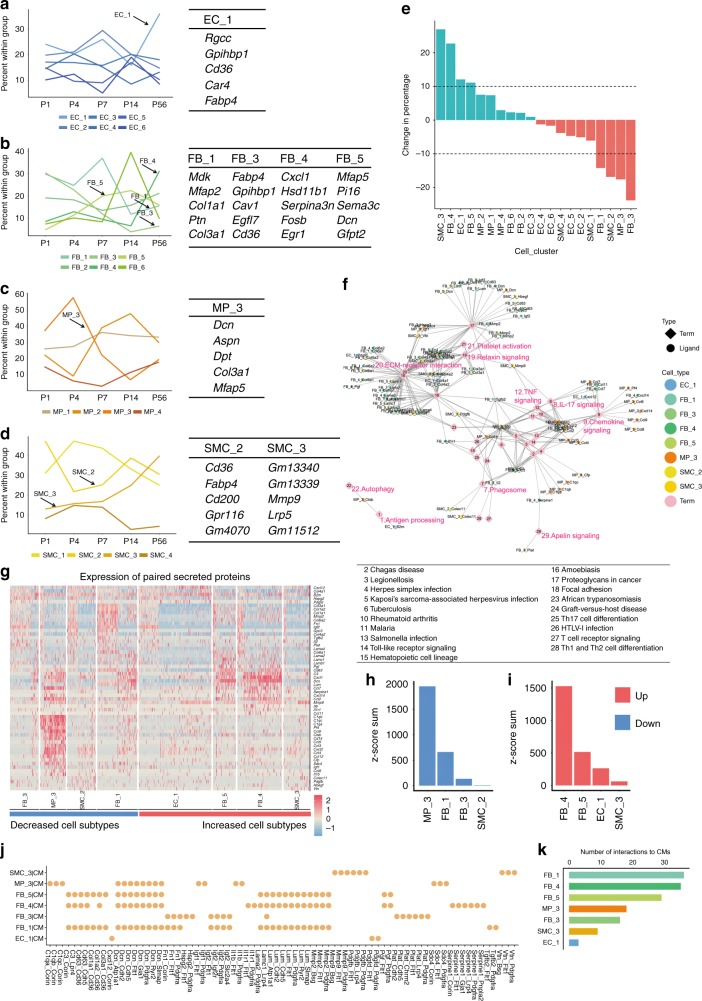
Prediction of cardiac fibroblasts as pro-maturation factor. **a**–**d** Subtype distribution of EC (endothelial cell, **a**), FB (fibroblast, **b**), MP (macrophage, **c**) and SMC (smooth muscle cell, **d**) across different stages. The total number of cells at each time point was taken as 100%. Arrows indicate cell subtypes with significantly changed proportions in P1 versus P56 hearts (see “Methods”). Right: selected top genes in cell subtypes apparently changed in P56 vs. P1 hearts, respectively. Please see Supplementary Data 8–11 for the full lists of genes in each cell subtype. **e** Ratio changes of altered cell clusters in P56 vs. P1. Dash lines indicate 10% cutoff which was used to define significantly changed cell subtypes in subsequent analyses (Supplementary Data 12). **f** Correlation analysis to show potentially matched pairs between the significantly altered genes encoding secreted proteins in representative cell clusters and signaling pathways in CM maturation during P1–P56 heart development. Each pink diamond represents signaling pathway (Supplementary Data 13). Each circle/dot indicates a secretory protein from a given cell type. Each connecting line indicates the correlation between a given secretory protein and a specific pathway. **g** Heatmap displaying the expression of genes in the correlation analysis (**f**) across all representative cell clusters (Supplementary Data 14). **h**, **i** Sum of all matched ligands (**f**) expressed in increased (**i**) or decreased (**h**) cell subtypes, respectively. **j** Bubble chart to show putative ligand–receptor pairs between differentially expressed receptors in CM clusters and corresponding ligands in significantly changed cell types (**g**). **k** Quantification of ligand–receptor pair counts (**j**) in each cell type.

**Fig. 3:**
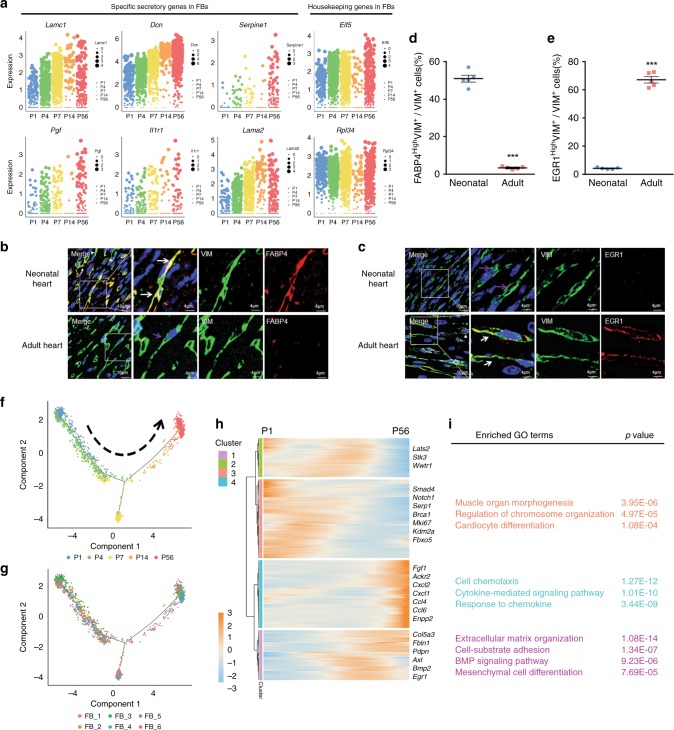
Subtype switching of cardiac fibroblasts highly correlates with CM maturation. **a** The expression of selected ligands potentially involved in CM maturation or housekeeping genes in FBs during maturation. **b**, **c** Immunofluorescent staining of VIM and FABP4 (**b**) or EGR1 (**c**) in neonatal (P1) or adult (P56) heart sections, respectively. White arrows indicate co-localization, purple arrows indicate VIM^+^ only cells. Scale bars = 10 or 4 μm. **d**, **e** Quantification of VIM^+^FABP4^High^ (**d**) or EGR1^High^ (**e**) cells in heart sections from P1 or P56 mice, Data are plotted as mean ± SEM, *n* = 5 biologically independent mice, ***p < 0.001, two-sided Student’s *t*-test. **f**, **g** Monocle analyses showing the ordering of FBs in pseudotime. Each color indicates either a time point (**f**) or a cell state (**g**). **h** Heatmap to display different blocks of top 1000 differentially expressed genes (DEGs) along the pseudotime trajectory (**f**, **g**). Please see Supplementary Data 17 for the full list. **i** Selected top gene ontology (GO) terms related to corresponding DEGs (Supplementary Data 18–20). Functional analysis was performed with enrichGO in clusterProfiler, *p* < 0.05 was considered significant enrichment. Source data are provided as a Source Data file.

Since non-cardiomyocytes (NCMs) form the niche for CMs, and actively modulate biological behaviors of the latter in heart development and disease, we sought to investigate how changes in the cellular microenvironment affect CM maturation. We partitioned major NCM cell types (including FB, EC, MP, and SMC) into subclusters, respectively (Supplementary Fig. 2a–h). Each cell type possessed multiple cell subtypes with distinct molecular features, and displayed dramatic shifts in their subtype compositions during heart maturation (Fig. 2a–d, Supplementary Data 8–11). To characterize alterations in the CM microenvironment between immature (P1) and mature (P56) hearts, and to understand how these differences contribute to CM maturation, we first identified cell subtypes whose proportions were significantly different between these two states (Fig. 2e, Supplementary Data 12). Then, we defined specific genes encoding secreted proteins in those altered NCM subtypes, and annotated these proteins to putative signaling pathways involved in CM maturation (Fig. 1k), which indicated the impacts of NCMs on CM maturation via ligands-based influences of related signaling pathways (Fig. 2f). Intriguingly, a number of secreted factors from multiple NCM subtypes were predicted to be involved in signaling pathways facilitating CM maturation (Fig. 2g). Of these cell subtypes, increased signals from fibroblast subtypes 4 and 5 (FB_4, FB_5), major FB subtypes in the adult (P56) heart, as well as decreased signals from MP_3 and FB_1, were predicted to play the most pronounced effects on signaling pathways related to cardiomyocyte maturation (Fig. 2h, i). Taking it a step further, we correlated secreted proteins released from top-ranked cell types (Fig. 2h, i) with CM receptors to ascertain their direct impact on cardiomyocyte maturation (Fig. 2j, Supplementary Fig. 2i, Supplementary Data 15). Congruently, FB subtypes (FB_1, FB_4, FB_5, and FB_3) possessed the highest number of secreted proteins potentially affecting CMs via ligand–receptor interactions (Fig. 2k). In addition, the expression of genes encoding potentially maturation-promoting proteins, including *Dcn*, *Lamc1*, *Pgf*, and *Lama2*, progressively increased in FBs over time, whereas the expression of housekeeping genes, such as *Eif5* and *Rpl34*, either remained unchanged or even moderately decreased (Fig. 3a, Supplementary Data 16). We next performed immunostaining against FABP4, a marker of FB_3 (subtype low in adult heart), or EGR1, a marker of FB_4 (subtype high in adult heart), with general fibroblast marker vimentin (VIM) to confirm fibroblast subtype switching from P1 to P56. In line with scRNA-Seq data, we observed substantial decrease in FB_3 (50.39% vs. 2.38% of all FBs), as well as increase in FB_4 (3.57% vs. 72.59% of all FBs), in P56-hearts, compared to P1-hearts (Fig. 3b–e). Next, we aligned all FBs in a pseudotime to recapitulate their development in vivo, and found that maturation of FBs were correlated with functions related to extracellular matrix organization, BMP signaling pathway, muscle organ morphogenesis, etc., which reinforced a possible role of FBs in CM maturation (Fig. 3f–i). Moreover, we applied SigHotSpotter to gain more insights into signaling pathways responsible for FB identity in P1 vs. P56^28^. While common signaling pathways related to fibroblast functions (e.g., focal adhesion, ECM-receptor interaction, etc.) were observed, FBs still displayed differential regulatory pathways/network in P1 (e.g., Foxo, mTOR, and VEGF, etc.) versus P56 (e.g., cell adhesion, estrogen, and TGF-β, etc.) (Supplementary Fig. 2j, Supplementary Data 21–24). Taken together, these observations prompted us to propose a model wherein fibroblast subtype switching from neonatal to mature state in postnatal heart development is a major driving force promoting CM maturation in vivo.

### Adult cardiac fibroblasts promote maturation of neonatal CMs

**Fig. 4:**
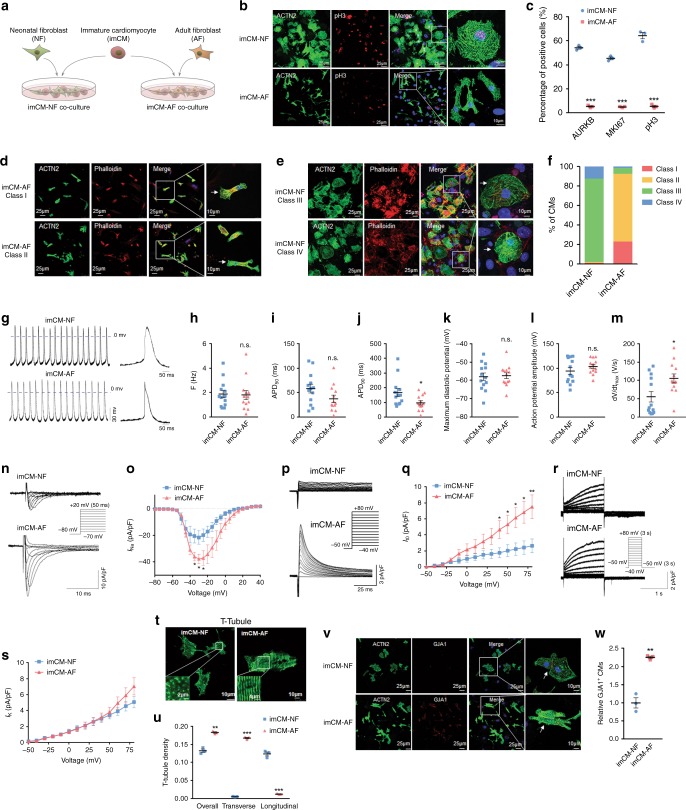
Adult cardiac fibroblasts promote maturation of neonatal CMs. **a** Schematic of in vitro co-culture of cardiomyocytes and cardiac fibroblasts. Freshly isolated immature CMs (imCMs) from P1 mouse hearts were immediately co-cultured with fibroblast monolayers from either neonatal (P1, NF) heart or adult (P56, AF) hearts, respectively. **b** Immunofluorescent staining against ACTN2 and pH3 in imCMs 3.5 days after co-culture. White arrows indicate cells with co-localization. Scale bars = 10 or 25 μm. **c** Quantification of ACTN2^+^AURKB^+^, ACTN2^+^MKI67^+^, and ACTN2^+^pH3^+^ cells 3.5 days after co-culture. **d**, **e** Immunostaining against ACTN2 and Phalloidin in co-cultures. Representative images to show typical class I–II (**d**) and class III–IV (**e**) CM morphology, respectively. **f** Quantification of **d**, **e**. **g** Representative action potential tracings of imCMs in co-culture. **h**–**m** Quantification of beating frequency (**h**), action potential duration to 50 and 90% repolarization (APD50, **i**; APD90, **j**), maximum diastolic potential (MDP, **k**), action potential amplitude (APA, **l**), and maximum rate of rise (d*V*/d*t*_max_) of action potential (**m**) of co-cultured imCMs. *n* = 13 cells. **n** Representative *I*_Na_ tracings of imCMs in co-culture with voltage control. **o** Quantification of **n**. *n* = 12 cells in imCM-NF = 13 cells in imCM-AF. **p** Representative *I*_to_ tracings of imCMs in co-culture. **q** Quantification of *I*_to_ (**p**) *n* = 11 cells. **r** Representative *I*_k_ tracings of imCMs in co-culture. **s** Quantification of *I*_K_ in **r**. *n* = 10 cells in imCM-NF = 15 cells in imCM-AF (**r**). **t** Representative images to show T-tubule structure in imCMs in co-culture. T-tubule was labeled by di-8-ANEPPS. **u** Quantification of T-tubule density in **t**. Image J was applied in quantification. **v** Immunofluorescent staining of ACTN2 and GJA1 in imCMs 3.5 days into co-culture. White arrows indicate co-localized cells. Scale bar = 10 or 25 μm. **w** Quantification of **v**. All data in this figure are plotted as mean ± SEM from three biologically independent experiments, *p < 0.05, **p < 0.01, ***p < 0.001, n.s. not significant, two-sided Student’s *t*-test. Source data are provided as a Source Data file.

To test our hypothesis, we applied an in vitro co-culture system, where immature cardiomyocytes (imCMs) isolated from neonatal (P1) hearts were co-cultured with either fibroblasts isolated from neonatal hearts (NF) or adult hearts (AF), respectively (Fig. 4a). Three and a half days into culture, the proportion of pH3^+^-imCMs (indicating proliferation) in NF co-culture remained 64.35%, whereas the proportion of proliferating imCMs in AF co-culture decreased to only 5.32% (Fig. 4b, c). Likewise, the percentages of AURKB^+^-and MKI67^+^-imCMs were also substantially lower in AF co-culture compared to NF co-culture (Supplementary Figs. 3a, b, 4c). These results suggested AF-induced cell cycle exit of imCMs, a sign of increased maturity. In addition to decreased proliferation, acquisition of mature cellular morphology and substructure is considered another hallmark of CM maturation, which can be classified into classes I–IV based on cellular morphology and filament alignment, corresponding to maturation states from high to low^29^. Consistent with little proliferation, imCMs co-cultured with AFs mostly displayed elongated morphology and well-arranged filaments (classes I and II), whereas those in co-culture with NFs exhibited round morphology with deranged or even fuzzy filaments (Fig. 4d–f, Supplementary Fig. 3c). Functional maturation of CMs is further marked by regular electrical activities, which are tightly controlled by an array of ion channels expressed on their plasma membranes. Therefore, we applied patch clamping to monitor action potentials of imCMs in co-cultures. Compared to NF-co-culture, imCMs co-cultured with AFs exhibited a decreasing trend in action potential duration to 50% repolarization (APD50) and to 90% repolarization (APD90), respectively, while beating frequencies remained similar (Fig. 4g–j). By contrast, action potential amplitude (APA) and maximum diastolic potential (MDP) remained unchanged, while maximum rate of rise (d*V*/d*t*_max_) of action potential was increased (Fig. 4k–m). In addition, AF co-culture significantly increased sodium currents (*I*_Na_) and transient outward potassium currents (*I*_to_), indicators of maturation, whereas calcium currents (*I*_Ca_) and delayed rectifier potassium current (*I*_K_) remained unchanged (Fig. 4n–s, Supplementary Fig. 3d–e). Further, AF co-culture significantly increased the calcium handling capacity of imCMs, which was also indicative of mature electrophysiology (Supplementary Fig. 3f–i). Despite an overall increasing trend, these observations suggested that electrophysiological maturation may be more sophisticated than molecular alterations in in vitro co-culture system.

Transverse tubules (T-tubules) are highly-specialized membrane extensions penetrating into the center of CMs, which is crucial in excitation–contraction coupling (ECC) by mediating the entering of electrical impulses into the cell. Thus, formation of T-tubule structure is considered a yet another hallmark of cardiomyocyte maturation^30^. Immunostaining showed that imCMs co-cultured with AFs exhibited T-tubule-like structures, which were less apparent in those co-cultured with NFs (Fig. 4t–u). Additionally, imCMs co-cultured with AFs showed increased expression of GJA1, a component of gap junctions, which are essential in the electrical conduction and synchronization of the adult heart (Fig. 4v–w). Collectively, these results demonstrated the strong capacity of AFs to promote maturation of imCMs in vitro.

### Molecular similarities of in vitro and in vivo CM maturation

**Fig. 5:**
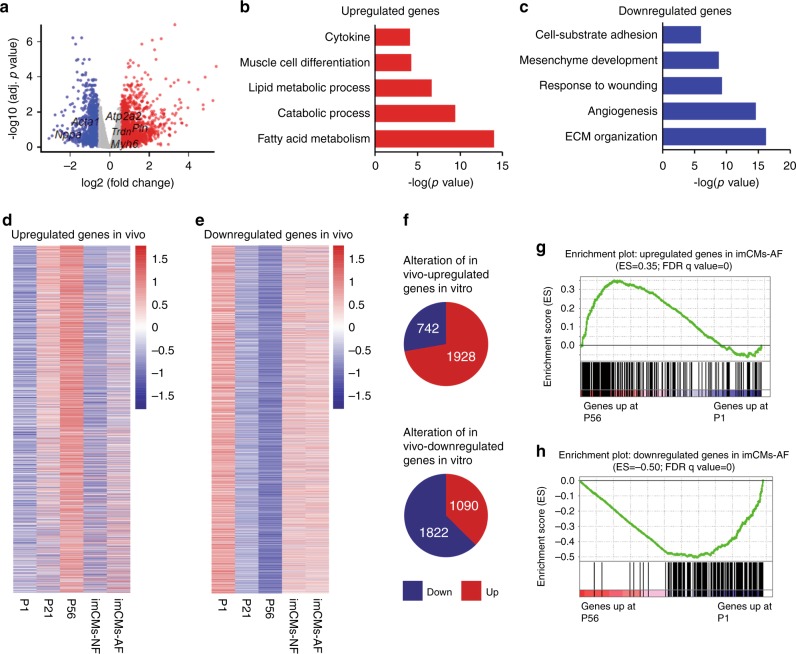
Molecular similarities of in vitro and in vivo CM maturation. **a** Volcano plot to show DEGs in imCMs 3.5 days after co-cultured with NFs or AFs. Red indicates upregulated genes, and blue represents downregulated genes upon AF co-culture compared to imCM-NF (Supplementary Data 25). **b**, **c** Gene ontology analyses of upregulated (**b**) or downregulated (**c**) genes from **a**. Selected top categories are shown. **d**, **e** Heatmaps to show the expression of upregulated (**d**) or downregulated (**e**) genes during postnatal heart maturation in imCMs co-cultured with NFs vs. AFs. Please see Supplementary Data 26 for the full list. **f** Top: number of genes upregulated or downregulated in vitro among upregulated genes in vivo (**d**); bottom: Number of genes upregulated or downregulated in vitro among downregulated genes in vivo (**e**). **g** Gene Set Enrichment Analysis (GSEA) showing that genes upregulated upon AF co-culture were enriched for genes that were highly expressed in P56 vs. P1 CMs. **h** Gene Set Enrichment Analysis (GSEA) showing that genes downregulated upon AF co-culture were enriched for genes that were highly expressed in P1 vs. P56 CMs.

To understand the molecular events giving rise to the observed morphological and functional maturation of imCMs co-cultured with AFs, we performed RNA-Seq on imCMs co-cultured with NFs (imCM-NF group) or AFs (imCM-AF group), respectively (Supplementary Fig. 3j). As a proof of concept, canonical immature cardiac genes, such as *Acta1*, were among the downregulated genes in imCM-AF, whereas genes related to CM contractility, including *Myh6* and *Pln*, were in the group of upregulated genes (Fig. 5a). Gene ontology (GO) analysis of genes that were upregulated upon AF co-culture showed significant enrichment in fatty acid metabolism and muscle cell differentiation, among others, which were dominant traits of mature CMs (Fig. 5b). By contrast, downregulated genes were significantly enriched in biological processes associated with extracellular matrix (ECM) organization, angiogenesis, etc (Fig. 5c). To further confirm a trend of maturation in imCMs co-cultured with AFs, we employed bulk RNA-Seq data of CMs isolated from P1 (immature), P21 (maturing), and P56 (fully mature) hearts as a reference dataset of differentially expressed genes (DEGs). Strikingly, genes upregulated during CM maturation in vivo also displayed a similarly increasing pattern in imCM-AF compared to imCM-NF (Fig. 5d). The same also applied to the set of downregulated genes during in vivo maturation, although to a lesser extent (Fig. 5e, f). In addition, GSEA showed that genes upregulated in imCMs-AF were drastically enriched in genes highly expressed in mature hearts (P56), whereas downregulated ones were significantly enriched in genes abundantly expressed in immature hearts (P1) (Fig. 5g, h). Together, these observations strongly suggested promotional effects of AFs on CM maturation at the molecular level.

### Identifying conserved signaling pathways in CM maturation

**Fig. 6:**
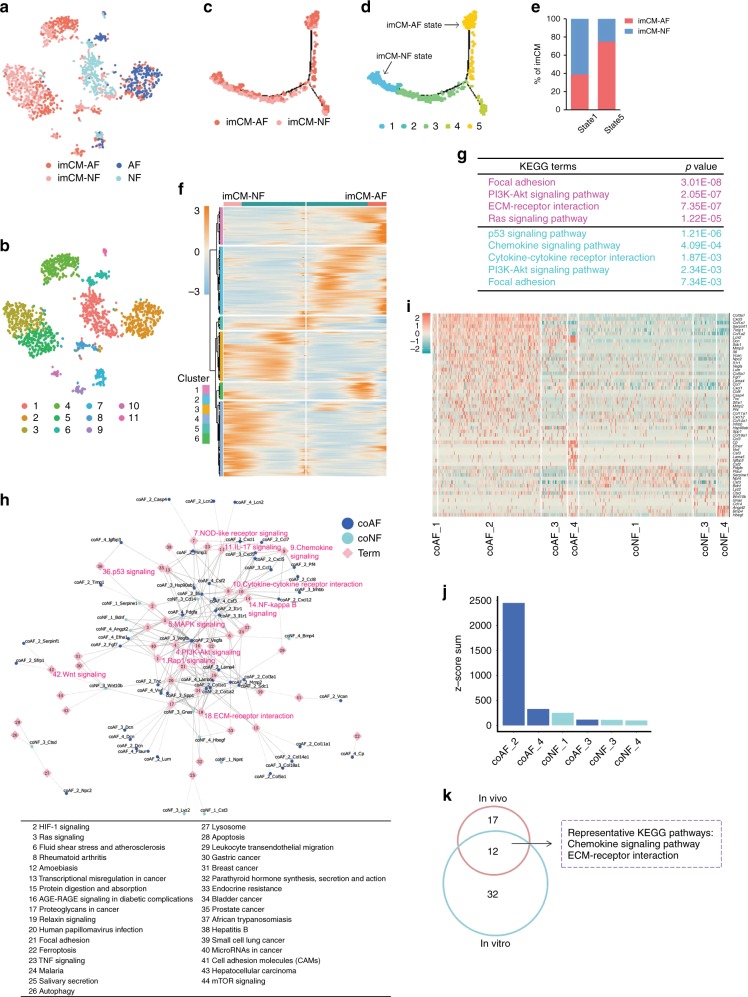
Identifying conserved signaling pathways in CM maturation. **a**, **b** *t*-SNE clustering of cells in both imCM-NF and imCM-AF co-culture systems by cell type (**a**) or cell subcluster (**b**). **c**, **d** Monocle analyses showing the ordering of imCMs co-cultured with NFs or AFs in pseudotime, marked by input cell type (**c**) or cell state (**d**). Arrows in **d** indicate representative state of imCM-NF or imCM-AF. **e** Percentages of imCM distribution in different cell states (**d**). **f** Heatmap to display differentially expressed genes (DEGs) along the pseudotime trajectory (**d**) (Supplementary Data 27). **g** Selected top Kyoto Encyclopedia of Genes and Genomes (KEGG) terms related to corresponding DEGs significantly changed during cardiomyocyte maturation in co-culture. Functional analysis was performed with enrichKEGG in clusterProfiler, *p* < 0.05 was considered significant enrichment. **h** Correlation analysis to show potentially matched pairs between the ligands secreted by representative cell clusters and signaling pathways in CM maturation (**g**) in the co-culture system. Each pink diamond represents signaling pathway (Supplementary Data 28). Each circle/dot indicates a secretory protein from a given cell type. Each connecting line indicates the correlation between a given secretory protein and a specific pathway. **i** Heatmap d**i**splaying the expression of genes indicated in correlation analysis (**h**) across all representative cell clusters (Supplementary Data 29). **j** Sum of all matched ligands (**i**) expressed in indicated cell clusters. **k** Venn diagram to show overlapping signaling pathways involved in cardiomyocyte maturation between heart development in vivo and in vitro co-culture (Supplementary Data 30). Representative 2 overlapping pathways are listed.

We next applied single-cell RNA sequencing (scRNA-Seq) to delineate the maturation trajectories of CMs in our co-culture system. A total of 1648 cells were sequenced, of which 1580 were included for further analysis after applying multiple filtering criteria (Supplementary Fig. 4a). We achieved a median sequencing depth of 178,652 reads/cell, 72% alignment rate/cell, and 2910 genes/cell (Supplementary Fig. 4b–d). Possible batch effects were ruled out by indistinguishable clustering using housekeeping genes (Supplementary Fig. 4e). Cells were defined as either CMs or FBs, based on their molecular signatures (Fig. 6a), which were further partitioned into 11 subclusters (Fig. 6b). To simulate the developmental route from an immature state (NF co-culture) to a mature state (AF co-culture), we aligned all CMs in pseudotime, which segregated into 5 distinct states (Fig. 6c, d). As anticipated, imCMs from the two groups displayed preferential distributions along the trajectory. In particular, whereas imCMs in co-culture with NFs were enriched in State 1, most imCMs co-cultured with AFs were localized in State 5 (Fig. 6c–e). Analyses of DEGs along the trajectory exhibited dramatic enrichment of the activation of key signaling pathways, including focal adhesion, ECM-receptor interaction, and chemokine signaling pathways (Fig. 6f, g). To gain overall insight into the contributions of fibroblasts to CM maturation, we constructed a putative correlation map between specific genes encoding secreted proteins in fibroblasts and signaling pathways implicated in CM maturation (Figs. 6h, i). In accordance with heart maturation in vivo (Fig. 2), EGR1^High^-fibroblasts (FB_4 in vivo and coAF_2 in vitro), mainly present in adult hearts, were predicted to play a predominant role in secreting proteins potentially promoting CM maturation (Fig. 6i, j, Supplementary Fig. 4f–g). Furthermore, to determine key pathways and secreted proteins highly conserved and critically important in CM maturation, we compared the profiles of in vivo and in vitro CM maturation, and observed significant overlap on predicted signaling pathways (12 out of 61), such as chemokine signaling pathway and ECM-receptor interaction, and ligands (18 out of 95), such as *Cxcl12* which was involved in overlapping pathways (Fig. 6k, Supplementary Fig. 4h). These observations indicated that the mechanisms AFs adopted to induce CM maturation in vitro closely resembled physiological conditions.

### Targeted inhibition of conserved pathways impairs maturation

**Fig. 7:**
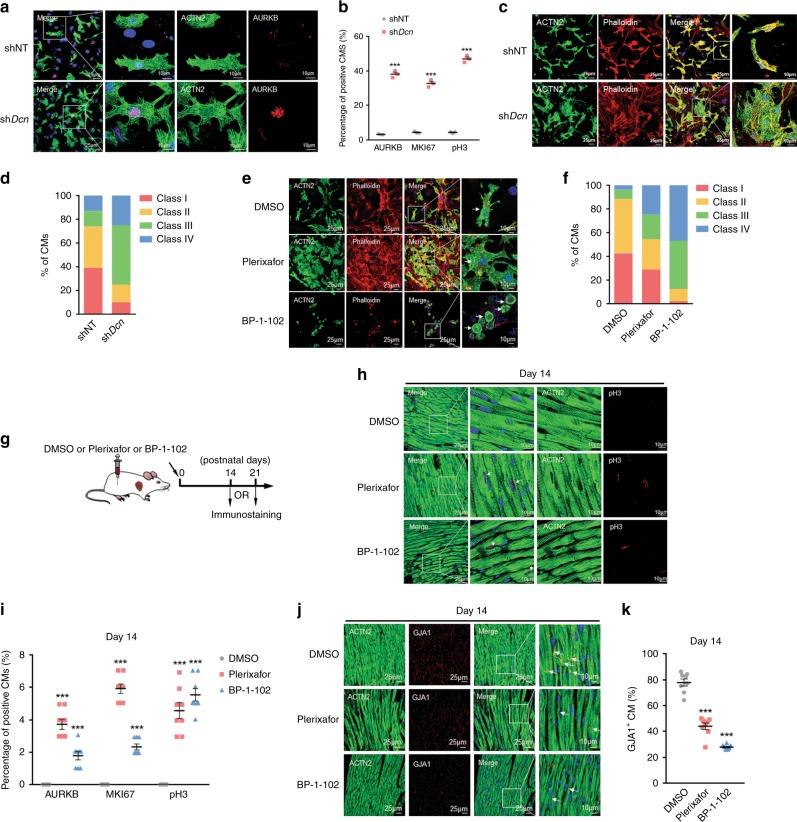
Targeted inhibition of conserved pathways impairs maturation. **a** Immunofluorescent (IF) staining against ACTN2 and AURKB in imCMs-AF upon transfection with shNT and sh*Dcn*, respectively. Scale bars = 25 or 10 μm. **b** Quantification of ACTN2^+^AURKB^+^ (**a**), ACTN2^+^MKI67^+^ (Supplementary Fig. 5b) or ACTN2^+^pH3^+^ (Supplementary Fig. 5c). Data are plotted as mean ± SEM from three independent experiments, ***p < 0.001, two-sided Student’s *t*-test. **c** Immunostaining against ACTN2 and Phalloidin in imCMs 3.5 days after co-culture with AFs upon depletion of NT (shNT) or *Dcn* (sh*Dcn*), respectively. **d** Percentages of CMs in different morphology grades in **c**. **e** Immunostaining against ACTN2 and Phalloidin in imCMs 3.5 days after co-culture with AFs in the presence of DMSO, Plerixafor or BP-1-102, respectively. **f** Percentage of CMs in different morphology grades in **e**. **g** Workflow of animal experiment. Neonatal (P1) mice were treated with DMSO, Plerixafor or BP-1-102 daily for 14 (Day 14) or 21 days (Day 21), respectively. Mice were sacrificed for immunostaining of heart sections at P14 or P21, respectively. **h** Immunofluorescent staining against ACTN2 and pH3 in heart sections from mice at P14. White arrows indicate co-localization. Scale bar = 10 or 25 μm. **i** Quantif**i**cation of ACTN2^+^AURKB^+^, ACTN2^+^MKI67^+^, and ACTN2^+^pH3^+^ cells^.^ Data are plotted as mean ± SEM, *n* = 8 biologically independent mice, ***p < 0.001, two-sided Student’s *t*-test. **j** Immunofluorescent staining of ACTN2 and GJA1 in heart sections of mice treated with DMSO, Plerixafor or BP-1-102 for 14 days, respectively. White arrows indicate co-localization. Scale bars = 10 or 25 μm. **k** Quantification of ACTN2^+^GJA1^+^ cells in **j**, Data are plotted as mean ± SEM, *n* = 8 biologically independent mice, ***p < 0.001, two-sided Student’s *t*-test. Source data are provided as a Source Data file.

To validate key molecules and signaling pathways in CM maturation, we first silenced secreted protein-encoding genes significantly involved in AFs-induced cardiomyocyte maturation in vitro (Supplementary Fig. 4h), such as *Dcn*, in AFs. As anticipated, silencing of *Dcn* in AFs significantly compromised AFs-induced CM maturation, characterized by preserved proliferation and lack of filament alignment (Fig. 7a–d, Supplementary Fig. 5a–c). Then, we sought to use inhibitors to target 2 signaling pathways on which multiple relevant genes converged (Fig. 6k). Drugs used included Plerixafor^31,32^, an antagonist for CXCR4 and CXCL12-mediated chemotaxis, to inhibit chemokine signaling pathway, and BP-1-102, a STAT3 inhibitor to suppress STAT3 phosphorylation-mediated synthesis of ECM^33^, as an ECM inhibitor to compromise ECM-receptor interaction. Consistent with silencing of individual proteins, inhibition of each of these two pathways severely compromised filament alignment of CMs (Fig. 7e, f), suggesting suppression of CM maturation. In the same vein, to uncover the importance of these pathways in vivo, we injected these 2 inhibitors into P1 neonatal mice, respectively, and monitored cardiomyocyte maturation at P14 and P21, respectively (Fig. 7g). Both Plerixafor and BP-1-102 treatment significantly preserved the proliferative capacity of CMs (AURKB^+^−, MKI67^+^−, and pH3^+^-CMs) compared to DMSO control on day 14 (Fig. 7h, i, Supplementary Fig. 6a, b), an effect that diminished on day 21 (Supplementary Fig. 6c–f). These results indicated that repression of these signaling pathways delayed cell cycle exit of CMs, which other mechanisms may compensate for over time. In parallel with temporarily reserved proliferative capacity, gap junction formation (GJA1 expression) was drastically compromised upon treatment with Plerixafor or BP-1-102 at both P14 and P21, respectively, a strong indication of retarded heart maturation (Fig. 7j, k, Supplementary Fig. 6g, h).

Given critical roles of the chemokine signaling pathway and ECM-receptor interactions in cardiomyocyte maturation, we sought to test the possibility of targeting them to manipulate CM maturity in the adult heart. To this end, we first treated adult mice with these 2 inhibitors, and evaluated cardiac function 2 weeks after treatment. In contrast to neonatal mice, CM proliferation, gap junction formation in CMs, as well as ejection fraction of hearts, remained similar across all groups, suggesting limited influence of these two pathways on cardiac function in normal adult hearts (Supplementary Fig. 7). Together, these observations indicated that the actions of these signaling pathways were specific to immature hearts.

### Pathway inhibition reverses CM maturation upon injury

**Fig. 8:**
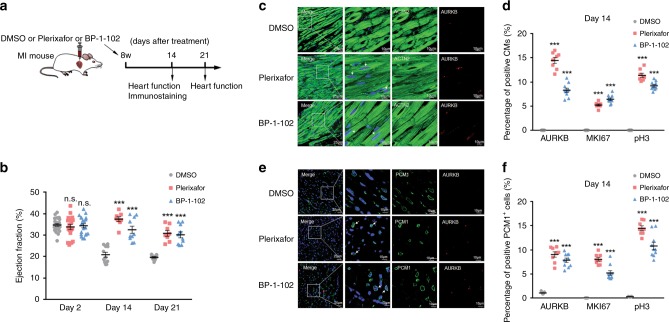
Pathway inhibition reverses CM maturation upon injury. **a** Workflow of animal experiment. Eight-week-old mice were subjected to myocardial infarction (MI) surgery, and corresponding inhibitors or DMSO were administered immediately afterwards. Mice were sacrificed 14 days post surgery, and hearts were removed for immunostaining. The additional set of mice was monitored for cardiac function on days 2, 14, and 21. **b** Quantification of ejection fraction based on echocardiogram. Data are plotted as mean ± SEM, *n* = 23, 18, 20 biologically independent mice in DMSO, Plerixafor and BP-1-102 group at Day 2, respectively, *n* = 11, 8, 10 biologically independent mice in DMSO, Plerixafor and BP-1-102 group at Day 14 and Day 21, respectively. ***p < 0.001, n.s. not significant, two-way ANOVA. **c** Immunofluorescent staining against ACTN2 and AURKB in the border zones of infarcts from (a). White arrows indicate co-localized cells. Scale bars = 10 or 25 μm. **d** Quantification of ACTN2^+^AURKB^+^ and ACTN2^+^MKI67^+^, and ACTN2^+^pH3^+^ cells^.^ Data are plotted as mean ± SEM, *n* = 11, 8, 10 biologically independent mice in DMSO, Plerixafor and BP-1-102 group, respectively. ***p < 0.001, one-way ANOVA. **e** Immunofluorescent staining against PCM1 and AURKB in the border zones of infarcts from **a**. White arrows indicate co-localized cells. Scale bars = 10 or 25 μm. **f** Quantification of PCM1^+^AURKB^+^ and PCM1^+^MKI67^+^, and PCM1^+^pH3^+^ cells^.^ Data are plotted as mean ± SEM, *n* = 11, 8, 10 biologically independent mice in DMSO, Plerixafor and BP-1-102 group, respectively. ***p < 0.001, one-way ANOVA. Source data are provided as a Source Data file.

We next tested the possibility to leverage such signaling pathways to promote CM proliferation upon cardiac injury. Specifically, we treated mice with Plerixafor or BP-1-102, or DMSO as vehicle control, immediately following myocardial infarction surgery (Fig. 8a). Intriguingly, both Plerixafor and BP-1-102 significantly improved cardiac function, and increased CM proliferation (AURKB^+^−, MKI67^+^−, and pH3^+^− CMs) in the border zones of infarcted areas, compared to DMSO control group (Fig. 8b–f, Supplementary Fig. 8). These results suggested that signaling pathways central to cardiomyocyte maturation may be exploited for the development of strategies to promote cardiac regeneration under stressed conditions.

### Conserved role of FBs in human cell model

**Fig. 9:**
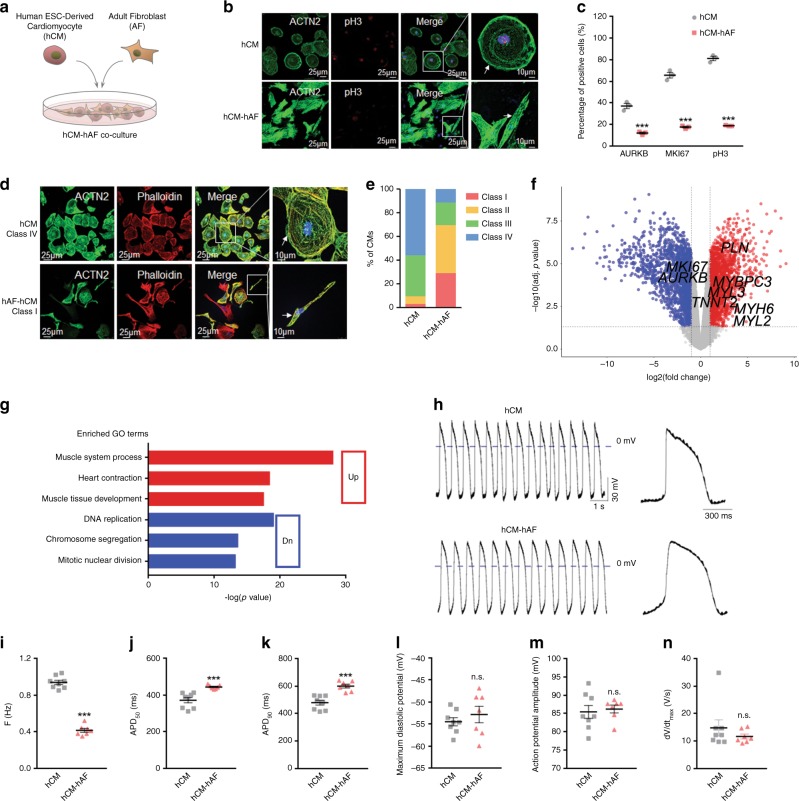
Conserved role of FBs in human cell model. **a** Schematic of co-culture of human cells in vitro. Cardiomyocytes induced from human embryonic stem cells (hESC-CMs) were co-cultured on a monolayer of cardiac fibroblasts isolated from adult human heart (hAFs). **b** Immunofluorescent staining against ACTN2 and pH3 in hESC-CMs 7 days after co-culture with or without hAFs, respectively. White arrows indicate co-localized cells. Scale bars = 10 or 25 μm. **c** Quantification of ACTN2^+^AURKB^+^, ACTN2^+^MKI67^+^, and ACTN2^+^pH3^+^ cells. **d** Immunostaining against ACTN2 and Phalloidin in the co-culture system. Representative images to show typical class I and class IV hESC-CM morphology when co-cultured with or without hAF, respectively. **e** Percentage of CMs in different morphology grades. **f** Volcano plot to show DEGs in hESC-CMs only versus those co-cultured with hAF. Red indicates upregulated genes, and blue represents downregulated genes upon AF co-culture. **g** Gene ontology analyses of upregulated or downregulated genes (**f**). Selected top categories are shown. **h** Representative action potential tracings of hESC-CMs alone or in co-culture with hAFs, respectively. **i**–**n** Quantification of beating frequency (**i**), action potential duration to 50% repolarization (APD50, **j**), to 90% repolarization (APD90, **k**), maximum diastolic potential (MDP, **l**), amplitude of action potential (APA, **m**), and maximum rate of rise (d*V*/d*t*_max_) of action potential (**n**) in (**h**). *n* = 8 cells in hCM, = 7 cells in hCM-hAF. All data in this figure are plotted as mean ± SEM from three biologically independent experiments, ***p < 0.001, n.s. not significant, two-sided Student’s *t*-test. Source data are provided as a Source Data file.

Human pluripotent stem cell-derived cardiomyocytes (hPSC-CMs) hold great promise for cardiac regenerative medicine. However, their immature properties remain a major hurdle in their clinical application. Given the predominant role of fibroblasts from adult mouse hearts in cardiomyocyte maturation, we asked whether human adult cardiac fibroblasts were also capable of promoting maturation of hPSC-CMs. To address this question, we isolated human adult cardiac fibroblasts (hAFs), and co-cultured them with human embryonic stem cell-derived CMs (hESC-CMs) (Fig. 9a). While most hESC-CMs were highly proliferative, as evidenced by high percentages of AURKB^+^−, MKI67^+^−, and pH3^+^− CMs, CMs co-cultured with hAFs exhibited marked decrease in their proliferative capacity (Fig. 9b, c, Supplementary Fig. 9a, b). In addition, co-culture with hAFs also significantly promoted morphological maturation of CMs (Fig. 9d, e, Supplementary Fig. 9c). At the transcriptional level, hAF co-culture significantly induced expression of cardiac genes, including *MYH6* and *TNNT2*, and repressed expression of proliferative genes, such as *MKI67* and *AURKB* (Fig. 9f). GO analysis of upregulated genes showed enrichment of biological behaviors related to muscle system process and heart contraction, whereas downregulated genes were significantly enriched in DNA replication and nuclear division, suggesting maturation of CMs (Fig. 9g). Noteworthily, Plerixafor and BP-1-102 failed to suppress co-culture-induced hESC-CM maturation, suggesting differential utilization of signaling pathways in AF-induced CM maturation in different species (Supplementary Fig. 9d–g).

In addition to molecular changes, hESC-CMs co-cultured with hAFs displayed increased APD50, APD90, in parallel with decreased beating frequency, as well as unchanged MDP, APA, and d*V*/d*t*_max_ compared to their hCM counterparts (Fig. 9h–n). These observations suggested a trend of electrophysiological maturation upon co-culture. To further validate enhanced functional maturation of hCMs in co-culture with hAFs compared to pure hCMs, we used calcium channel blocker nifedipine to assess their responses with respect to extracellular field potential (EFP), impedance, and beating rate, and observed significant changes in these parameters in the hCM-hAF, but not in the hCM group (Supplementary Fig. 9h–j). Collectively, these findings demonstrated that hAFs promoted maturation of hCMs in vitro, suggesting relatively conserved impacts of the cellular environment of cardiomyocyte maturation, which potentially offers strategies of promoting maturation of hESC-CMs in culture.

## Discussion

It is well established that the cellular microenvironment profoundly affects cell behaviors in various physiological and pathological contexts, including stem cell fate decision and cancer progression^34,35^. However, how alterations in the cellular microenvironment contribute to cardiac maturation lack systematic illustration. In this study, we analyzed scRNA-Seq data to gain insight into molecular events occurring in both cardiomyocytes (CMs) and non-cardiomyocytes (NCMs) during postnatal development in mice, and identified crucial cellular interactions and regulatory signaling networks. In-depth analysis of cellular crosstalk identified hitherto unknown contributions of certain NCM subtypes to cardiomyocyte maturation, of which fibroblasts from the adult heart demonstrated the most pronounced effects on relevant signaling pathways.

Cardiac fibroblast is a crucial NCM cell type in the heart, and is recognized to play complex roles in normal and dysfunctional hearts. For example, cardiac fibroblasts were reported to induce hypertrophic responses in cardiomyocytes by secreting factors like TGF-β and FGF-2 in response to pressure overload^36,37^. By contrast, other studies determined cardiac fibroblasts as the major cell source of interleukin (IL)-33 production, which exerted antihypertrophic effects to the same stimulus^38,39^. These seemingly controversial functions of fibroblasts may be the net outcome of highly context-dependent and dynamic compositions of fibroblast subpopulations, each of which endowed with slightly different functionalities. Therefore, heightened awareness of the different properties of cardiac cell subtypes and their dynamics may yield insight into previously unexplained phenomena or irreconcilable controversies.

Cardiac maturity is central to heart development and disease. It is generally accepted that progressive dedifferentiation of cardiomyocyte, and thus loss of CM identity, is the hallmark molecular event in cardiac hypertrophy^40,41^. By contrast, much less is understood about its microenvironmental setting, especially with regard to how the microenvironment transforms in disease, and subsequently translates into deteriorations in CMs. Our findings of the critical role of fibroblast subclusters in promoting CM maturation may extend into future work determining their role in the maintenance of CM maturity in the normal heart, as well as their changes and relationship with CM dedifferentiation in cardiac hypertrophy.

Another commonly encountered heart disease is myocardial infarction (a.k.a. heart attack), where blood flow to the myocardium is abruptly cut off, thereby causing tissue damage. Given the limited regenerative capacity of CMs, developing techniques to regenerate the damaged myocardium remains the major hurdle in the prognosis of such patients. Previous studies have demonstrated that at the border zone of infarcted regions, existing CMs dedifferentiate into an immature state, with a parallel increase in proliferation^42^. Although incapable of fully repairing injury, these observations provide critical clues on the regeneration of damaged myocardium, namely, via proliferation of endogenous CMs. Therefore, the key to cardiac repair became finding a way to induce already matured CMs into a relative immature state with high proliferative potential. Given our observations that adult fibroblasts facilitated cardiomyocyte maturation, one might speculate on their potential inhibitory roles in cardiomyocyte proliferation/dedifferentiation, and thus in cardiac damage repair. In line with this idea, inhibition of key signaling pathways involved in adult fibroblast-induced cardiomyocyte maturation significantly increased CM proliferation both in postnatal development and myocardial infarction, suggesting that these cells may be a potential target to manipulate CM fate in physiology and disease. Noteworthily, inhibitors used in our study broadly target key signaling pathways in all cell types, rather than CMs only, raising the possibility that improved cardiac function or CM proliferation was due to indirect effects on other cell types. Indeed, previous studies reported that CXCR4 inhibition, as the pharmacological effect of Plerixafor treatment, promoted cardiac repair and improved outcomes via T cell mobilization and recruitment of bone marrow progenitor cells for angiogenesis^43,44^. Therefore, CM-specific targeting of these signaling pathways may be required to test their roles in CM proliferation in vivo in the future.

In addition to adult fibroblasts, subclusters of other cell types, including MP_3, SMC_3, and EC_1 in the adult heart, were also predicted to regulate cardiomyocyte maturation, although to a much lesser extent. Interestingly, a recent study uncovered a critical role of subtype switching of macrophage in progression of cardiac hypertrophy^45^. In addition, ACKR1^+^ EC was revealed to play a central role in maintenance of adult human heart function^46^. Therefore, it is worth exploring how other cell (sub)types exert their effects on cardiac maturation and other biological aspects in future studies. Moreover, identification of pertinent key signaling pathways may provide potential strategies to manipulate CM fate in postnatal heart development and cardiac diseases.

Stem cell-based, especially pluripotent stem cell-based, therapies hold great promise in cardiac regenerative medicine. However, adverse effects owing to their incomplete maturation prohibited their effective translation into the clinic^1,4^. Inspired by our single-cell analysis, we co-cultured immature CMs (imCMs) with adult fibroblasts in vitro to mimic cellular interactions in vivo, which, as anticipated, significantly enhanced maturity of CMs. Most importantly, human ESC-CMs attained advanced maturation traits upon co-culture with cardiac fibroblasts from adult human heart. These observations highlighted a central role of cardiac fibroblasts in CM maturation. Interestingly, a previous study suggested a critical role of embryonic fibroblasts in embryonic CM proliferation^23^, highlighting fibroblasts as major constituents of the microenvironment regulating CM fate. Thus, it may seem paradoxical to strive for high purity in the induction of cardiomyocytes from pluripotent stem cells, as is the goal of current methodologies, when cells actually yearn for a much more sophisticated niche for full maturation. Given minimal effects on maturation through prolonged culture of PSC-CMs, we may need to reconsider our current strategies to achieve better CM maturation in vitro^47^. Indeed, several efforts were put into promoting CM maturation by mimicking in vivo circumstances, such as 3D culture, physical conditioning, and engineered cardiac tissues using non-cardiac fibroblasts^26,27,48–50^, underscoring the importance of delineating the cellular microenvironment surrounding CMs. To this end, our study unbiasedly scrutinized postnatal heart maturation at single-cell resolution. In addition to a panoply of genes, pathways, and cell subtypes identified, we unveiled FB subtype switching as a critical microenvironmental event endogenously regulating postnatal cardiomyocyte maturation. Findings from our study not only offer ways to better reconstitute the natural niche to induce maturation of stem cell-derived CMs, and to minimize unwanted effects associated with stem-cell based therapies, but also shed light on how to leverage related proteins and pathways to manipulate CM fate in development and diseases.

## Methods

### Animals and human tissue samples

All animal protocols were approved by the Institutional Animal Care and Use Committee, Experimental Animal Center, Fuwai Hospital, National Center for Cardiovascular Diseases, China. All mice were housed in standard SPF condition with temperatures of 65–75 °F (~18–23 °C) and with 40–60% humidity. Eight-to-ten-week-old male C57BL/6 mice were used in this study unless otherwise specified; the number of mice studied was indicated for each experiment respectively. Mice were randomly assigned into groups by means of drawing lots. Echocardiographic analyses were performed by an independent investigator blinded to the procedure. For the use of human tissue, informed consent was obtained from subjects in the clinic for use of the human heart tissue in research.

### Drug treatment of animals

P1 C57BL/6 mice and their mothers were purchased from the National Institutes for Food and Drug Control and used for evaluating the effect of Plerixafor and BP-1-102, respectively. Mice were administrated 5 mg/kg Plerixafor, 3 mg/kg BP-1-102 or DMSO through subcutaneous injection every 24 h for 14 or 21 days, respectively. By the end of treatment, cardiac function was determined via echocardiography (VisualSonicsVeVo 2100 Imaging System) by evaluating ejection fraction (EF) and left ventricular fractional shortening (FS). Additionally, hearts were immediately harvested after perfusion, fixed with 10% formalin, and embedded in paraffin for immunofluorescence staining. Adult mice received drug treatment following the same protocol.

### Myocardial infarction model

Chronic myocardial ischemia was achieved by permanently ligating the left anterior descending (LAD) coronary artery. Briefly, 8-week-old C57BL6J mice were anesthetized with isoflurane, subjected to chest hair removal, and ventilated with a volume-cycled rodent respirator with 1–2 ml per cycle at a respiratory rate of 120 cycles per min. Next, thoracotomy was performed at the third intercostal space and self-retaining microretractors were placed to separate the third and fourth rib to visualize the LAD. A 7-0 prolene suture (LINGQIAO, Ningbo medical needle Co., Ltd) was then passed under the LAD at 1 mm distal to the left atrial appendage. The LAD was doubly ligated. Successful ligation was confirmed by color loss of the LV wall. Next, the chest wall was closed with a 7-0 prolene suture (LINGQIAO, Ningbo medical needle Co., Ltd) and the skin was closed with 4-0 prolene suture (F404, Shanghai PudongJinhuan Medical Supplies Co., Ltd.). Finally, mice were extubated and allowed to recover from surgery under a heating lamp. Sham-operated mice underwent the same procedure but without ligation. The mouse surgeon was blinded to the study. At day 14, cardiac function and ventricular structure were determined via echocardiography (VisualSonicsVeVo 2100 Imaging System). At the end of the study (day 21), hearts were harvested, and prepared for immunofluorescence staining.

### Isolation of murine cardiomyocytes and cardiac fibroblasts

Neonatal hearts were resected from C57BL/6J mice on postnatal 1 day, and cardiomyocytes were digested using the Pierce™ Primary Cardiomyocyte Isolation Kit according to the manufacturer’s protocol (Thermo, #88281). In brief, left ventricles freshly dissected from neonatal hearts were minced into 1–3 mm^3^ pieces in a 1.5 ml sterile microcentrifuge tube, and 0.2 ml of reconstituted Cardiomyocyte Isolation Enzyme 1 (with papain) and 10 µl Cardiomyocyte Isolation Enzyme 2 (with thermolysin) were added to each tube, mixed gently and incubated at 37 °C for 30–35 min. Then, tissues were washed with 0.5 ml of Complete DMEM for Primary Cell Isolation, triturated, and centrifuged at 1000 rpm at room temperature. Finally, cells were filtered through a 100 µm-cell strainer to remove undigested tissues and to obtain single-cell suspension. Resulting single cells were plated onto a 10 cm dish for differential velocity adherence for 70 min, at which point non-cardiomyocytes (NCMs) mainly including neonatal fibroblasts (NFs) adhered, whereas cardiomyocytes (CMs) did not, and thus could be isolated.

Adult mouse hearts were dissociated by retrograde perfusion with enzymatic Tyrode’s solution supplemented with 1 mg/ml collagenase II (Sigma) on a Langendorff perfusion apparatus as previously described^51^. In brief, adult mouse were anesthetized by isoflurane, and retrogradely perfused for about 15 min at a constant flow of 4 ml/min with Tyrode’s buffer at 37 °C containing (mM): 120 NaCl, 1.2 MgCl_2_, 10 KCl, 1.2 KH_2_PO_4_, 10 glucose, 10 HEPES, 20 taurine, 5 pyruvate, adjusted to pH = 7.4 with NaOH. Left ventricles were excised and cut into small pieces in enzyme-free buffer containing 1 mg/ml BSA and gently dissociated for 1 min to facilitate myocyte dissociation. Cell suspensions, which mainly were adult fibroblasts (AFs), were incubated for another 2 min and filtered through a 100 µm-cell strainer to remove undigested tissues.

All isolated FBs were cultured for three passages to increase their purity before co-culture. Both neonatal and adult fibroblasts were subsequently cultured in fibroblast medium (10% FBS in DMEM) for the following co-culture experiments.

### Isolation of adult human cardiac fibroblasts

Adult human heart tissues were resected from donors from the clinic, and fibroblasts were digested using the Pierce™ Primary Cardiomyocyte Isolation Kit according to the manufacturer’s protocol (Thermo, #88281) as mentioned above. In brief, freshly dissected human hearts were minced into 1–3 mm^3^ pieces in a 1.5 ml sterile microcentrifuge tube, and 0.2 ml of reconstituted Cardiomyocyte Isolation Enzyme 1 (with papain) and 10 µl Cardiomyocyte Isolation Enzyme 2 (with thermolysin) were added to each tube, mixed gently and incubated at 37 °C for 30–35 min. Then, tissues were washed with 0.5 ml of Complete DMEM for Primary Cell Isolation, triturated, and centrifuged at 1000 rpm at room temperature. Finally, cells were filtered through a 100 µm-cell strainer to remove undigested tissues and to obtain single-cell suspension. Resulting single cells, mainly including adult fibroblasts (hAFs), were plated onto a 10 cm dish for culturing, and the medium will be changed every day. Fibroblasts were subsequently cultured in fibroblast medium (10% FBS in DMEM) for the following co-culture experiments.

### Single-cell RNA-Seq

Cells in co-cultures, stained by CellTracker^TM^ Green CMFDA or CellTracker^TM^ Res CMTPX, were digested with 0.25% trypsin for 1 min at 37 °C, rigorously pipetted up and down several times, filtered through a 100 μm-cell strainer, and subjected to FACS sorting. Single-cell sequencing libraries were generated using the ICELL8 platform (Takara). In brief, isolated cells were stained with a mixture of Hoechst 33342 and Propidium Iodide (Thermo, R37610) according to the manufacturer’s instruction. After washing with PBS, cells were counted on the Moxi^TM^ Automated Cell Counter. A cell suspension of 20,000 cells/ml was submitted to the MultiSample NanoDispenser (MSND, Wafergen Biosystems) for single cell preparation. Dispensed cells were then imaged on the Imaging Station, and single live cells, defined by Hoechst-positive and Propidium Iodide-negative staining, were selected. Selected cells were subjected to reverse transcription and first-step amplification on a Chip Cycler (Bio-Rad), and the resulting cDNA was purified and size-selected with Agencourt AMPure XP beads (A63880, Beckman Coulter)^52^. One ng of purified cDNA was used to generate a sequencing library with the Nextera XT DNA sample preparation Kit (Illumina, FC-131-1024). Libraries were sequenced on the NextSeq 500 sequencer (Illumina) using the 26nt and 50nt paired-end sequencing protocol.

### Cell co-culture system in vitro

Immature cardiomyocytes (imCMs) were co-cultured with neonatal cardiac fibroblasts (NFs, plating ratio 1:0.2) at postnatal day 1 (P1) or with adult cardiac fibroblasts (AFs, plating ratio 1:0.33) from C57BL/6J mice for 3.5 days before endpoint assessments. Relatively fewer neonatal fibroblasts were used to accommodate their proliferation. imCMs co-cultured with fibroblasts were maintained in DMEM supplemented with 10% FBS.

Human embryonic stem cells (H1) were plated on Matrigel-coated plates (Corning), and maintained in TeSR™-E8™ media (StemCell Technologies, Cat. #05990). A monolayer-based directed differentiation protocol was used to generate hESC-CMs^53^. In brief, on day 1 of differentiation, hESCs were induced by changing the culture media to RPMI1640 containing 3 µM CHIR-99021HCl (SelleckChem, Cat. #S2924), and 2% B-27 minus insulin (Gibco). After 3 days of culture, the media was replaced with RPMI1640 containing 5 µM IWR-1-endo (SelleckChem, S7086) and 2% B-27 minus insulin (Gibco) for 2 days. On day 5, the media was again changed to RPMI1640 containing 2% B-27 minus insulin (Gibco) for 3 days. From day 8 onward, cultures were fed every 3 days with RPMI1640 plus 1× B-27 supplement. For human cardiac co-culture, human ESC-derived cardiomyocytes were co-cultured with adult human cardiac fibroblasts (hAFs) at a ratio of 1:0.33 (1.33 × 10^5^ cells in total in one well of 6-well plate) in DMEM supplemented with 10% FBS for 7 days.

### Lentivirus-based gene silencing

shRNA sequences were designed as follows: sh*Dcn*: CCTGTCTAAGAACCAACTAAA. shRNA were cloned into the pLKO.1 lentivirus-based vector, and lentivirus was packaged according to manufacturer’s instructions. Adult cardiac fibroblasts or neonatal cardiomyocytes were transduced with corresponding lentiviruses at approximately 80% confluency, and co-culture experiments were performed 2 days after transduction, respectively. Subsequent analyses were conducted 3.5 days after co-culture.

### Immunofluorescence staining

Cells were grown on glass coverslips in the presence of gelatin, fixed for 15 min in cold 4% paraformaldehyde (PFA), and incubated with 5% normal goat serum (NGS; ZSGB-BIO, Beijing, China) in PBS with 1% Triton X-100 (Sigma) for 1 h at room temperature. Primary antibodies were incubated overnight (over 18 h) at 4 °C, protected from light. After further incubation at 37 °C for 45 min, cells were incubated with corresponding secondary antibodies conjugated with FITC488 or Alexa Fluor 594 in the dark, and mounted with fluorescent mounting medium. Images were taken on a confocal scanning laser microscope (Leica, SP8, Germany).

Immunostaining with anti-ACTN2 (1:200, ab9465, Abcam, USA), anti-TNNT2 (1:100, ab8295, Abcam, USA), and anti-PCM1 (G-6) (1:50, sc-398365, Santa Cruz, USA) were used as cardiomyocyte markers. To examine cardiac proliferation, the following antibodies were used: monoclonal anti-AURKB antibody (1:100, ab2254, Abcam), anti-MKI67 antibody (1:100, ab15580, Abcam), anti-phospho-histone (pH3) antibody (1:100, 53348, CST). To examine cardiomyocyte maturation, the following antibodies were used: anti-GJA1 antibody (1:100, 15386-1-AP, proteintech), EGR1 antibody (1:100, 55117-1-AP, proteintech), FABP4 antibody (1:100, ab92501, Abcam). To examine fibroblast identity, the following antibodies were used: anti-VIM antibody (ab193555, Abcam), anti-VIM antibody (ab8978, Abcam). The TRITC-Phalloidin reagent (1:300, Thermo Fisher) was used to visualize F-actin for morphology assessment.

The secondary antibodies used in this study were fluorescein (FITC488)-conjugated goat anti-mouse IgG (H + L) (1:400, ZA-0511, ZSGB-BIO, China) and Alex Fluor 594-conjugated goat anti-rabbit IgG (H + L) (1:400, ZA-0512, ZSGB-BIO, China). FITC488-conjugated goat anti-rabbit IgG (H + L) (1:400, ZA-0513, ZSGB-BIO, China), Alex Fluor 594-conjugated goat anti-mouse IgG (H + L) (1:400, ZA-0514, ZSGB-BIO, China), and FITC488-conjugated goat anti-rat IgG (H + L) (1:400, ZA-0515, ZSGB-BIO, China). Cell nuclei were counterstained with 0.1% 4′,6-diamidino-2-phenylindole (DAPI, ZSGB-BIO, China).

Definition of cardiomyocyte classes was based on the following criteria (morphology and filament arrangement): Class I, elongated CMs with clear and well-arranged filaments; Class II, elongated CMs with less clear or well-arranged filaments; Class III, round-shaped CMs with some clear filaments; Class IV, round-shaped with ambiguous filaments. For quantification in vitro, six randomly chosen fields in each group per experiment, and values were plotted as mean ± SEM from five independent experiments. For quantification in vivo, six randomly chosen fields in each section, and five sections from each mouse.

### Flow cytometry

Adult cardiac fibroblasts (passages 3–6) and neonatal imCMs (P1), from C57BL/6J mice were stained by CellTrackerTM Green CMFDA (Thermo, C2925) at 37 °C for 20 min, respectively, and imCMs were stained with CellTrackerTM Res CMTPX (Invitrogen, C34552) at 37 °C for 20 min. Co-cultures were established as described above. The medium was renewed every day. All samples were digested by 0.25% trypsin and analyzed on the BD FACS Canto^TM^ II flow cytometry system (BD Biosciences). Only cells with one fluorescence dye (green or red) were collected thereafter.

### Cardiomyocyte T-tubule staining

imCMs were isolated and co-cultured as previously described. Samples were incubated with di-8-ANEPPS (10 μM, Thermo, D3167) for 10 min in a black cassette at room temperature. T-tubules were visualized using a Leica TCS SP8 confocal scanning microscope (Leica, Germany). T-tubule density was determined by thresholding to mean fluorescence intensity of the entire cell using ImageJ. The t-tubule density was then calculated for the interior of the cell, which was defined as the above-threshold area divided by the cross-sectional area. Transverse and longitudinal t-tubule quantities were normalized to cell area and t-tubule density of individual cells.

### Ca^2+^ transient measurements

For measurements of Ca^2+^ transients, the cells were loaded with fluo-4, AM (20 μM, Thermo, F14217) for 30 min at 37 °C in the dark and superfused with a 37 °C HEPES Tyrode solution. Spontaneous action potentials or calcium waves were recorded every 3 ms using a Leica TCS SP8 STED confocal scanning microscope (Leica, Germany), with a 512 pixel line drawn along the longitudinal axis of the cell. Data were acquired using ImageJ and were quantified as the extracellular background signal subtracted from fluorescence intensity (*F*) and then normalized to the baseline fluorescence (*F*0). Transient amplitude was presented as a relative scale (Δ*F*/*F*0).

### Electrophysiological recording

Whole-cell patch clamping was applied for ionic current and action potential recordings with an Axon 200B patch-clamp amplifier (Axon Instruments, USA). Ionic currents were recorded with Giga-Ohm seal in voltage clamp mode, and action potentials were recorded in current clamp mode. Pipette resistances were 1.5–3 MΩ. Current is expressed as current density (normalized cell capacitance). Data were recorded with sampling rates up to 2.0 kHz.

For action potential recording, the external solution contained (in mmol/l): 140 NaCl, 4 KCl, 2 CaCl_2_, 1 MgCl_2_, 10 glucose, and 10 HEPES, pH 7.4 with NaOH. Pipette solution contained (in mmol/l): 120 KCl, 1 MgCl_2_, 3 MgATP, 10 Hepes, and 10 EGTA, pH 7.2 adjusted with KOH.

For calcium current recording, the external solution contained (in mmol/l): 135 NaCl, 4 KCl, 2 CaCl_2_, 1 MgCl_2_, 10 glucose, and 10 HEPES, pH 7.4 with NaOH. Pipette solution contained (in mmol/l): 110 CsCl, 6 MgCl_2_, 5 Na_2_ATP, 10 HEPES, and 15 TEA-Cl, pH 7.2 adjusted with CsOH. tetrodotoxin (0.02 mmol/l) and 4-aminopyridine (5 mmol/l) were added to inhibited *I*_Na_ and *I*_to_. Calcium current was elicited by 1-s depolarizing steps from a holding potential of −70 mV to potentials ranging from −70 to +60 mV in 10-mV increments.

For *I*_to_ and *I*_K_ recording, tetrodotoxin (0.02 mmol/l), nifedipine (0.01 mmol/l), and BaCl_2_ (0.5 mmol/l) were added to blocked *I*_Na_, *I*_Ca_, and *I*_K1_, respectively. The external solution contained (in mmol/l): 140 NaCl, 4 KCl, 2 CaCl_2_, 1 MgCl_2_, 10 glucose, 10 HEPES, pH 7.4 with NaOH. Pipette solution contained (in mmol/l): 130 KCl, 10 HEPES, 5 MgATP, 10 EGTA, and 1 MgCl_2_, pH 7.2 adjusted with KOH. *I*_to_ current was elicited by depolarizing steps from a holding potential of −50 mV to potentials ranging from −40 to +80 mV in 10-mV increments. *I*_to_ was measured as the transient component of the outward current before the development of *I*_K_. *I*_K_ current was elicited by 3 s depolarizing steps from a holding potential of −50 mV to potentials ranging from −40 to +80 mV in 10 mV increments, followed by a repolarization to −50 mV.

To record *I*_Na_, we used an external K^+^-free solution that contained (in mmol/l):117.5 CsCl, 20 NaCl, 1 CaCl_2_, 1 MgCl_2_, 10 glucose, and 20 HEPES, pH 7.4 adjusted with CsOH. Pipette solution contained (in mmol/l): 110 CsCl, 6 MgCl_2_, 5 Na_2_ATP, 10 HEPES, and 15 TEA-Cl, pH 7.2 adjusted with CsOH. *I*_Na_ current was elicited by 100-ms depolarizing steps from a holding potential of −80 mV to potentials ranging from −80 to +40 mV in 5 mV increments. The interval between voltage steps was 3 s.

### Contractility and extracellular field potential measurement

The impedance of hESC-derived CMs were measured with CardioExcyte96 (Nanion Technologies) to study pharmacological effects on contractility and coordinated ion channel activity through extracellular field potential (EFP) recordings.

For human cardiac co-culture, cells were co-cultured with adult human NCMs in DMEM media supplemented with 10% FBS. Before experimentation, cells were pelleted and then resuspended in DMEM media to yield a cell density of 40,000–60,000 viable cells per well on the NSP-96 to ensure the formation of a synchronously beating monolayer. All hESC-CMs used in this study were monitored during spontaneous beating using the CardioExcyte Control software (Nanion Technologies GmbH).

Nifedipine (300 nM, Sigma-Aldrich), which is an L-type Ca^2+^ channel blocker causing QT shortening in cardiomyocytes, was prepared at 2× concentration in culture media, and used at a final concentration of 1×. Measurements of impedance and EFP were taken every 5 min for a length of 20 s, over a period of 2 h.

Data were analyzed using DataControl 96 (Nanion Technologies GmbH). In general, a mean beat was generated in impedance and EFP modes by aligning individual beats and calculating mean beat and standard deviation.

### RNA-Seq

FACS-sorted CMs and NCMs were pelleted by centrifugation at 300 × *g* for 3 min. Total RNA was extracted with the GeneJET RNA Purification Kit (Thermo, K0732). In brief, 600 µl of lysis buffer supplemented with β-mercaptoethanol was added to cell pellets, and mixed thoroughly by pipetting, followed by addittion of 360 µl ethanol (100%), after which the sample was mixed again. The sample was transferred to the GeneJET RNA Purification Column inserted in a collection tube to centrifuge the column for 1 min at ≥12,000 × *g*, followed by serial washes: 700 µl Wash buffer 1600 µl of Wash buffer 2 and 250 µl of Wash buffer 2. Finally, 100 µl of nuclease-free water was added to elute RNA. Sequencing libraries were prepared using the TruSeq RNA Library Prep Kit V2 (RS-122-2002, Illumina) according to manufacturer’s instructions. All libraries were sequenced on the NextSeq500 sequencer (FC-404-2005, Illumina) using the 35nt paired-end sequencing protocol.

### Single-cell RNA-Seq quality control

Raw base call (BCL) files were converted to FASTQ files using bcl2fastq (2.18.0.12). Raw reads were processed in four steps: (1) Only read pairs whose read 1 uniquely mapped the pre-defined barcode tag (10 nt) and UMI (14 nt) were considered as valid. (2) Read pairs were filtered by cutadapt (v1.8.1). The parameters were: -m 20 --trim-n --max-n 0.7 –q 20. 3) Reads were then aligned to genomes of *E. coli*, mycoplasma, yeast, and adapter sequences by bowtie2 (v2.2.4)^54^. Contaminants were filtered by FastQ Screen (v0.5.1.4). Clean reads were then mapped to the UCSC mm10 genome via STAR (v2.5.2b)^55^ and assigned to Ensembl genes^56^ by featureCounts. Sequencing reads were further filtered and sorted by a custom filtering pipeline (see below).

For cell filtering, the number of captured transcripts per gene was inferred based on UMIs, and all read pairs with UMIs containing Ns were excluded. Only cells with a minimally detected gene number of 500 were kept for downstream analysis^52^. To remove background signal, only genes detected in at least ten cells were considered as passing the threshold. In addition, reads mapped to all 37 mitochondrial genes were excluded (Supplementary Data 32). Then, cells were filtered with genome alignment percentage over 50%, and distinct UMI number less than 1e6, and between the average distinct UMI number ±2* standard deviation across all the cells. Further, as illustrated by Butler et al.^57^, per-gene transcript counts were normalized across cells. Within each single cell, the UMI count of each gene was divided by total UMIs of that cell, and multiplied by size factor 10,000 to obtain a TPM-like value, which was then transformed to natural logarithm. A total of 1648 cells were isolated from co-cultures, and 7507 cells from mice, both reaching an average raw read depth of ~0.1 M reads per cell. After filtering, data of 1580 co-cultured cells and 6368 freshly isolated cells passed the criteria described above.

### Single-cell clustering and annotation of cell clusters

Clustering was conducted using the Seurat package (v2.1.0)^57^. Firstly the most variable genes among cells were selected by their average expression level and dispersion level: average log TPM-like value was restricted to be between 0.01 and 3, and the dispersion was set to be greater than 1. Then, the selected genes were used for cell clustering. The FindClusters function of Seurat based on shared nearest neighbor (SNN-cliq) clustering method was utilized with default parameters (*k* = 30). FACS-sorted co-cultured CMs and FBs were clustered as described above. The first ten PCA components were used to do SNN clustering at a resolution of 1. Each cluster was annotated as either CM or FB according to the expression of known markers. To preclude misclassification, only cells identified as the same cell type by both FACS and cell clustering were kept for analysis. For downloaded data (mouse P1 to adult), to remove any potential systematic batch effect in the data, canonical correlation analysis (CCA) was applied to identify cell clusters by RunMultiCCA function in Seurat. Each chip was considered a batch of experiment, and both number of UMI and number of genes in each cell were used as regression factors.

To address whether there was systematic batch effect in our data, we selected 107 house-keeping genes including 37 tRNA genes, 36 transcription elongation factor genes, 19 RNA polymerase II subunit genes, and 15 ribosomal genes (Supplementary Data 33). Then, a single-cell experiment object was constructed by scater (1.10.1), SingleCellExperiment (1.4.1) and scran (1.8.4). PCA analysis on these genes showed that all groups were mixed, and no obvious distinct pattern was observed. Moreover, any cluster in *t*-SNE analysis comprising cells from a single animal or replicate was considered an outlier cluster, and therefore was removed from downstream analysis. In our *t*-SNE results, all clusters contained cells from multiple animal samples or replicates. Hence, the difference among cell clusters is considered true biological differences rather than systematic technical issues.

### Identification of variable genes

The FindAllMarkers function was used to identify variable genes^57^, the with parameters test.use = wilcox, min.pct = 0.2, thresh.use = 0.2, only.positive = TRUE for clarifying the identity genes of each group of cells, and FALSE for identifying the DEGs between multiple groups of cells. Genes were filtered with a *q* value of 0.05. For block differential expression analysis, such as comparing CMs and other cells, all subclusters were combined. For differential expression analysis in a subgroup of cells, each subgroup was compared with all other cells.

### Cell trajectory analysis

Monocle2 (v2.6.0)^58^ was used to study pseudotime trajectories of cells. The UMI matrix was used as input and variable genes detected by Seurat were used for building traces. Branches in the cell trajectory represent cells that have alternative gene expression patterns.

### Cell type contribution analysis in cardiomyocyte maturation

To evaluate the contribution of different cell types, we performed the following steps: (1) We collected all signaling pathways potentially involved in CM maturation based on Monocle analysis. (2) We identified non-cardiomyocyte cell subtypes with significant proportion changes (using 10% as cutoff) in P56 (mature) compared to P1 (neonatal, immature) hearts, whose changes might be involved in CM maturation. (3) We next hypothesized that those cell subtypes whose proportions changed with maturation affected the signaling pathways in CM maturation via ligand–receptor interaction. (4) Then, we identified top-ranked secreted proteins specifically expressed in these cell subtypes (Please see “Methods” below), and retrieved KEGG pathways associated with these secreted proteins using program kg. Further, overlapping pathways between retrieved KEGG pathways and CM maturation-related signaling pathways were collected and shown in a network. (5) Upon such correlation, we evaluated the expression of all the linked ligands in the changed cell subtypes. (6) We ranked the overall impact of cell subtypes by summing all annotated ligands. (7) We next evaluated whether CMs expressed corresponding receptors to interact with those annotated ligands. To do this, we identified receptors expressed on CMs (please see “Methods” below), and paired them with those ligands according to the protein-protein interaction library constructed from STRING database (see “Methods” below). (8) Finally, we quantified ligand–receptor pairs to assess the contribution of each cell type to CM maturation.

### Cell–cell interaction network analysis

Mouse secreted proteins were extracted from the UniProt database^59^, with keywords: locations:(location:“Secreted [SL-0243]”) AND reviewed:yes AND organism: “Mus musculus (Mouse) [10090]”. A total of 1503 genes were retrieved as secreted genes. Membrane protein keywords: annotation:(type:transmem) goa:(“plasma membrane [5886]”) AND reviewed:yes AND organism: “Mus musculus (Mouse) [10,090]”. A total of 1509 genes were retrieved as membrane genes. We used FindAllMarkers function of Seurat to define specifically expressed genes in each cluster (*q* value < 1e−3). Genes encoding secreted protein were selected as possible ligands. Cell clusters with a change over 10% from P1 to P56 were considered significantly changed, and were kept for cell–cell interaction network analysis. To discover possible secreted proteins that directly influence change of CM from P1 to P56, we used a cross-match method. Firstly the KEGG pathways associated with the selected secreted proteins of each cluster were retrieved by the program kg. Program kg is a Python CLI and API that enables retrieval of KEGG pathways, their definitions and their related genes. It is an open-source software released on GitHub. Then the pathways were overlapped with the enrichment result of branching DEGs in the CM trajectory from P1 to P56. Only overlapping terms and associated ligands were kept. Further, a network graph of those functional terms and ligands were drawn using R package network (v1.13.0.1), sna (v2.4) and GGally (v1.4.0), and a heatmap of those associated secreted proteins were generated in Seurat.

To evaluate the total secreting capacity of each cell clusters. The sum of scaled expression (*z*-score) of all specific secreted proteins of each cell cluster was calculated, and the cell clusters were ordered by their *z*-score sum of all specific secreted proteins.

### Ligand–receptor interaction analysis

A protein–protein interaction library was retrieved from the STRING database (10090.protein.links.full.v10.5.txt)^60^, including 3,616,945 interacting pairs with experimental evidence. For each time point, CM-specific genes were identified by comparing CM cells to the rest of cells, using the FindMarkers function. Then, CM-specific membrane receptors were selected. The selected secreted proteins of each NCM cluster were retrieved by program kg as described above. Further, all potential pairs of ligand–receptor interactions were identified, according to a pre-built protein–protein interaction library. Then a circular layout was produced to visualize all those interactions, using circlize (0.4.6), and each line in the plot indicates a pair of ligand–receptor interaction. The number of ligands to CM receptors was summed up in each cell cluster.

### Bulk RNA-sequencing analysis

For RNA-Seq analysis of both co-cultured cells and mouse P1 to adult RNA-Seq data downloaded, reads were firstly aligned to genomes of mm10 by subread (R version 1.24.2)^61^. Unique reads were kept and then assigned to in-build refseq gene annotation of subread using featureCounts. Genes were further filtered, and only those with RPKM > 1 in two or more than two samples were kept for differential analysis^62^. Differential analysis was conducted with limma^63^. Genes with adjusted *p* value < 0.05 and absolute logFC > 1 were taken as significantly differentially expressed genes in P1 to P56 data and hCM co-culture data, and adjusted *p* value < 0.05 and absolute logFC > 0.58 for co-culture data.

### Statistics and reproducibility

All results are expressed as means ± SEM. Student’s *t*-test was used for comparison of two groups, one-way ANOVA, post hoc or two-way ANOVA was used for comparison of three groups in the manuscript, *p < 0.05, **p < 0.01, ***p < 0.001, n.s., not significant. *p* < 0.05 was considered statistically significant. All representative images or results were selected from at least three independent experiments with similar results.

### Reporting summary

Further information on research design is available in the Nature Research Reporting Summary linked to this article.

## Supplementary information

Supplementary Information

Reporting Summary

Description of Additional Supplementary Files

Supplementary Data 1

Supplementary Data 2

Supplementary Data 3

Supplementary Data 4

Supplementary Data 5

Supplementary Data 6

Supplementary Data 7

Supplementary Data 8

Supplementary Data 9

Supplementary Data 10

Supplementary Data 11

Supplementary Data 12

Supplementary Data 13

Supplementary Data 14

Supplementary Data 15

Supplementary Data 16

Supplementary Data 17

Supplementary Data 18

Supplementary Data 19

Supplementary Data 20

Supplementary Data 21

Supplementary Data 22

Supplementary Data 23

Supplementary Data 24

Supplementary Data 25

Supplementary Data 26

Supplementary Data 27

Supplementary Data 28

Supplementary Data 29

Supplementary Data 30

Supplementary Data 31

Supplementary Data 32

Supplementary Data 33

## Data Availability

The authors declare that all data supporting the findings of this study are available within the article and its supplementary information files or from the corresponding author upon reasonable request. Raw sequencing data generated in this study have been deposited at the GEO database under accession code: GSE123547. Single-cell RNA-Seq data of mouse cardiomyocytes in postnatal P1 to P14 have been deposited in the GEO database under accession code: GSE122706 and were generated in a completely separate study by our group using the same single-cell platform as in this study, and all are publicly available. The source data underlying Figs. 3d–e, 4c, h–m, o, q, s, u, w, 7b, i, k, 8b, d, f, 9c, i–n, and Supplementary Figs. 3e–i, 5a, 6f, h, 7b–c, f–g, 8a–d, 9e, j are provided as a Source Data file.

## Source data

Source Data

## Code availability

Corresponding codes can be accessed from GitHub using the following link https://github.com/LiWangLab/Cardiomyocyte_Maturation.
