# Supplementary material for: Single-cell analysis of murine fibroblasts identifies neonatal to adult switching that regulates cardiomyocyte maturation
Source: Nat Commun. 2020 May 22;11:2585. doi: 10.1038/s41467-020-16204-w (PMC7244751; doi:10.1038/s41467-020-16204-w)
Supplement: Supplementary file 1 — Supplementary Information [file 41467_2020_16204_MOESM1_ESM.pdf]

Single cell analysis of murine fibroblasts identifies neonatal to adult switching which regulates cardiomyocyte maturation

Wang et al.

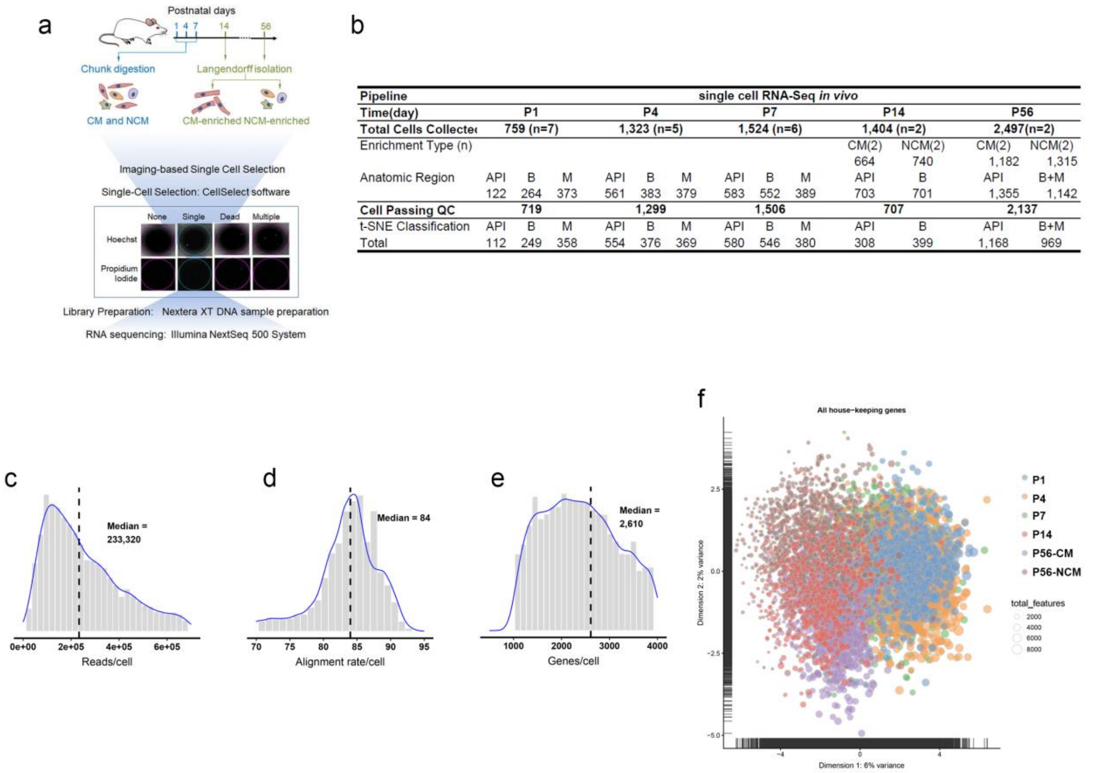

Pipeline	single cell RNA-Seq *in vivo*												
Time(day)		P1			P4			P7		P14		P56	
Total Cells Collecte(		759 (n=7)			1,323 (n=5)			1,524 (n=6)		1,404 (n=2)		2,497(n=2)	
Enrichment Type (n)										CM(2)	NCM(2)	CM(2)	NCM(2)
										664	740	1,182	1,315
Anatomic Region	API	B	M	API	B	M	API	B	M	API	B	API	B+M
	122	264	373	561	383	379	583	552	389	703	701	1,355	1,142
**Cell Passing QC**		719			1,299			1,506		707		2,137	
t-SNE Classification	API	B	M	API	B	M	API	B	M	API	B	API	B+M
Total	112	249	358	554	376	369	580	546	380	308	399	1,168	969

Supplementary Figure 1. Single-cell analysis of murine postnatal heart development. (a) Schematic of study design. Cells were isolated from mouse hearts of postnatal day 56 (P56) using the Langendorff method. Single cells were imaged and selected using the CellSelect Software. cDNA libraries of single, live cells were prepared and sequenced on the Illumina NextSeq 500 sequencer. Single-cell data of hearts at other time points were downloaded from Gene Expression Ominbus (GSE122706). CM, cardiomyocyte; NCM, non-cardiomyocyte. **(b)** Animal information and numbers of cells of P1, P4, P7, P14, and P56 mice processed via single-cell RNA sequencing. AR, apex region; BR, border region next to apex; RR, remote region away from apex; QC, quality control. **(c-e)** Quality metrics for single-cell RNA-Seq data showing distributions of number of reads per cell (c), alignment rate per cell (d), and number of genes detected per cell (e). **(f)** Principal component analysis (PCA) of all collected cells using 107 housekeeping genes.

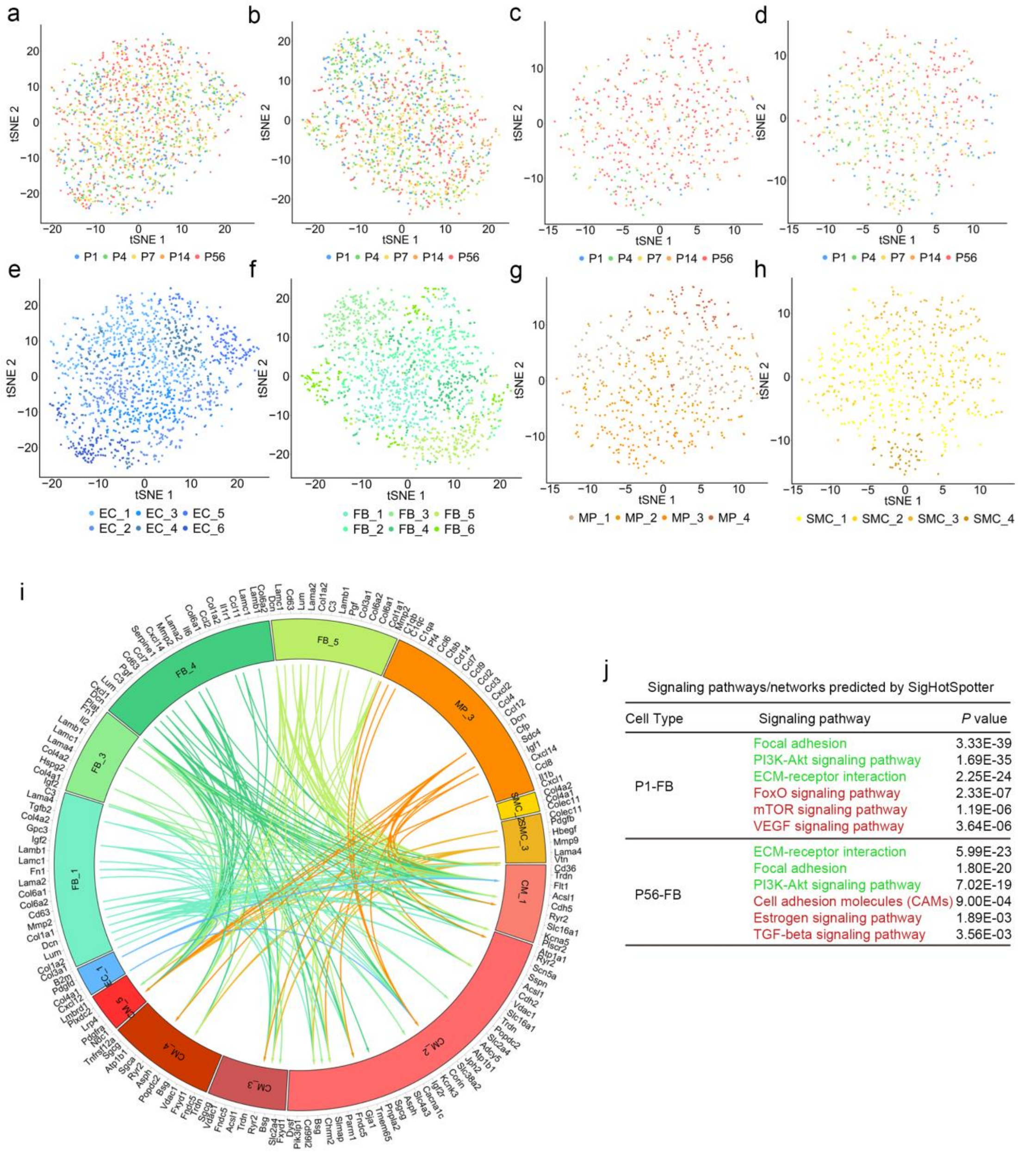

j

Signaling pathways/networks predicted by SigHotSpotter

Cell Type	Signaling pathway	*P* value
P1-FB	Focal adhesion	3.33E-39
	PI3K-Akt signaling pathway	1.69E-35
	ECM-receptor interaction	2.25E-24
	FoxO signaling pathway	2.33E-07
	mTOR signaling pathway	1.19E-06
	VEGF signaling pathway	3.64E-06
P56-FB	ECM-receptor interaction	5.99E-23
	Focal adhesion	1.80E-20
	PI3K-Akt signaling pathway	7.02E-19
	Cell adhesion molecules (CAMs)	9.00E-04
	Estrogen signaling pathway	1.89E-03
	TGF-beta signaling pathway	3.56E-03

Supplementary Figure 2. Prediction of cardiac fibroblasts as pro-maturation factor. (a-h) *t*-SNE clustering of EC (a, e), FB (b, f), MP (c, g), and SMC (d, h) from different time points. Each color indicates either a time point (top) or a cell cluster (bottom). **(i)** Putative ligand-receptor pairs between differentially expressed receptors in CM clusters and corresponding ligands in top-ranked cell types (Fig. 2g). Please see Supplementary Data 15 for the full list. **(j)** Predicted signaling pathways/networks responsible for FB identities in P1 and P56 hearts, respectively. Green represents signaling pathways shared in both types of

fibroblasts, whereas Red indicates differential pathways. Functional analysis was performed with enrichKEGG in clusterProfiler, $p < 0.05$ was considered significant enrichment. Please see Supplementary Data 20-24 for the full lists.

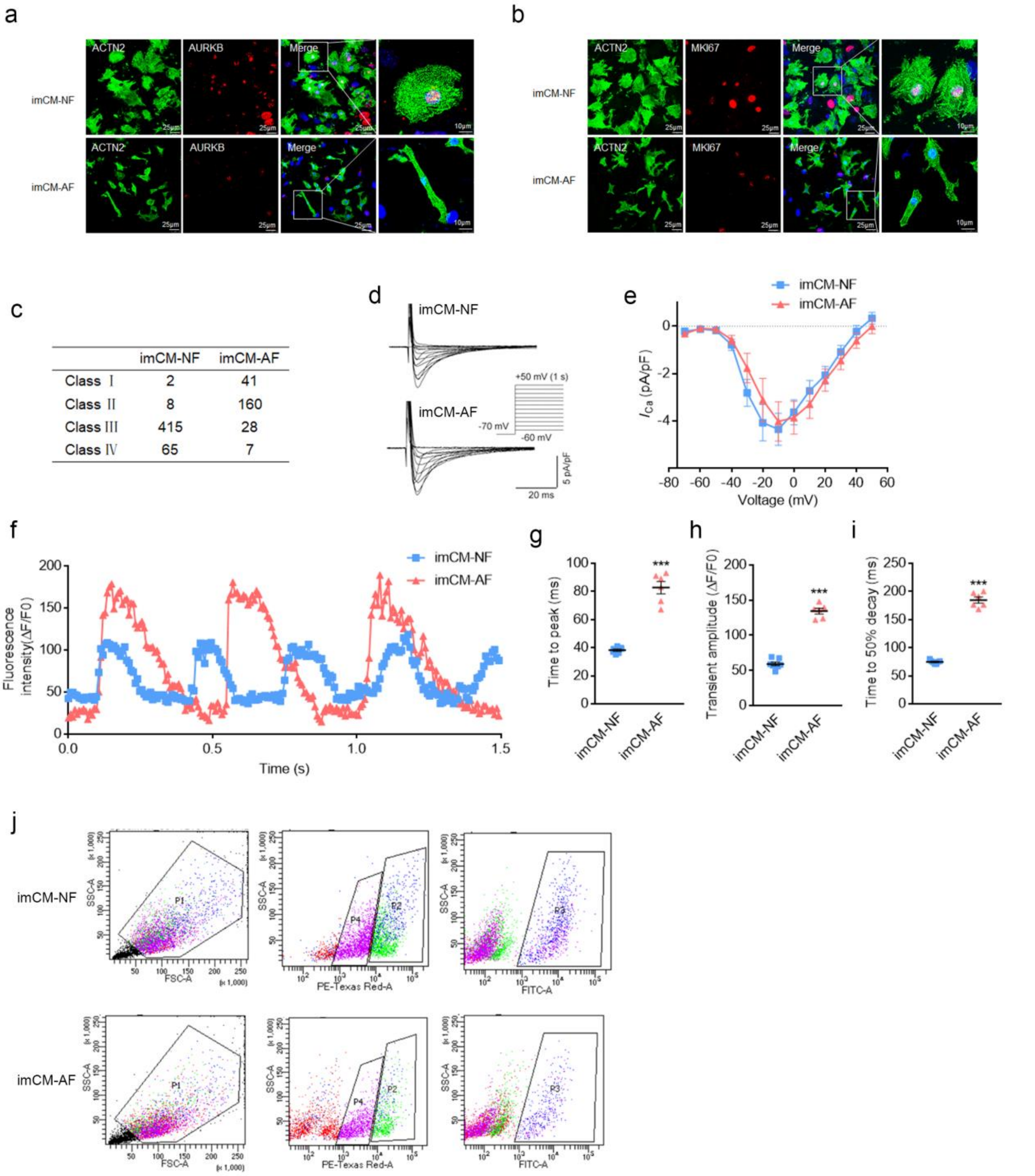

Supplementary Figure 3. Adult cardiac fibroblasts promote maturation of neonatal CMs.

(a-b) Immunofluorescent (IF) staining against ACTN2 and AURKB (a) or MKI67 (b) in imCMs 3.5 days after co-culture with NFs or AFs, respectively. Scale bar = 10 or 25 μm. **(c)** Percentage of CMs in different morphology grades upon co-culture with NFs or AFs, respectively. **(d)** Representative I_{Ca} tracings of imCMs in co-culture. **(e)** Quantification of (d). Data are plotted as mean ± SEM, n = 18 cells in imCM-NF, = 16 cells in imCM-AF examined over 3 independent experiments, two-sided Student's *t*-test. **(f)** Representative tracings of Ca^{2+}

transients in imCMs co-cultured with NF or AF, respectively. **(g-i)** Quantification of time to peak (g), transient amplitude (h), and time to 50% decay (i) in (f). Data are plotted as mean ± SEM, n = 7 cells in imCM-NF, = 6 cells in imCM-AF examined over 3 independent experiments, ***p < 0.001, two-sided Student's t-test. **(j)** Gating strategies used to collect corresponding cells after co-culture for bulk RNA-Seq (Fig. 5). P1 indicates live cells, P2 (CellTracker™ Red CMTPX) indicates imCMs co-cultured with NFs or AFs, P3 (CellTracker™ Green CMFDA) indicates NFs (top) or AFs (bottom). Source data are provided as a Source_Data_Extended_Data_Fig. 3.

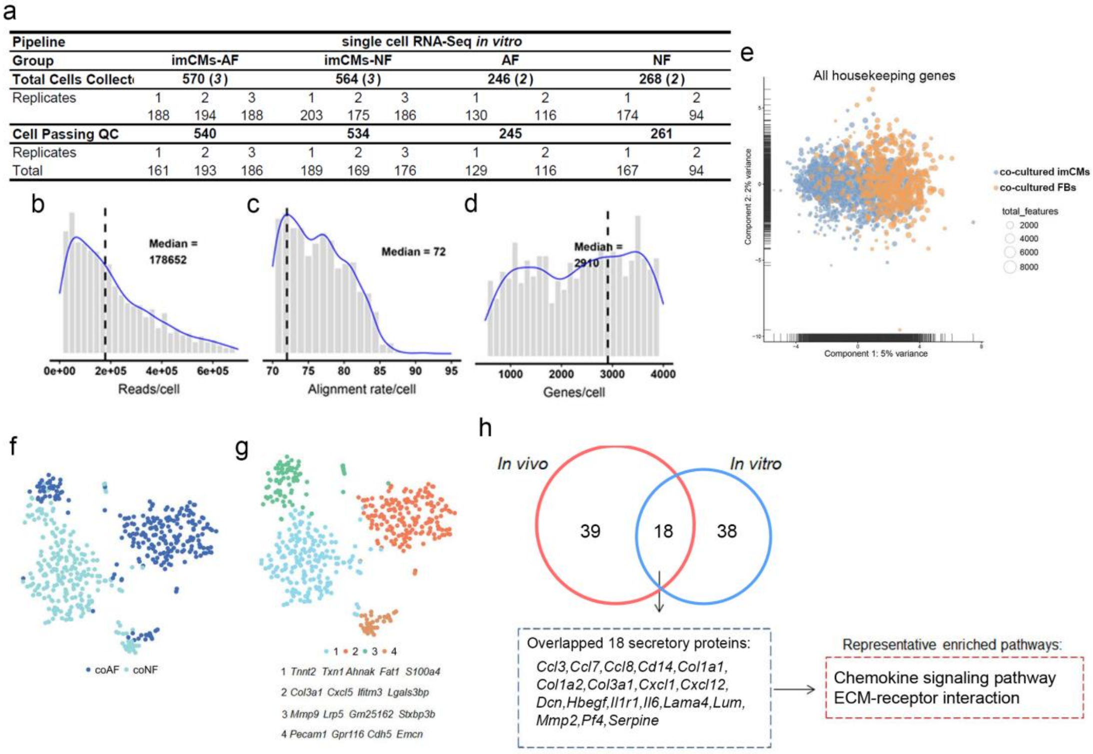

Supplementary Figure 4. Identifying conserved signaling pathways in CM maturation. **(a)** Numbers of cells in co-culture system processed via single-cell RNA sequencing. imCMs-AF, immature CMs co-cultured with AF; imCMs-NF, immature CMs co-cultured with NF; AF, fibroblast from adult (P56) heart in co-culture system; NF, fibroblast from neonatal (P1) heart in co-culture system; QC, quality control. **(b-d)** Quality metrics for single-cell RNA-Seq data showing distributions of number of reads per cell (b), alignment rate per cell (c), and number of genes detected per cell (d). **(e)** Principal component analysis (PCA) of all collected cells using 107 housekeeping genes. **(f-g)** *t*-SNE clustering of FBs from the co-culture system. Each point represents a single FB, while each color indicates either the source of FB (f) or a cell cluster (g). Right, top 5 markers in each FB subcluster. **(h)** Venn diagram to show overlapping ligands indicated in cardiac maturation between heart development *in vivo* and co-culture system *in vitro*. All 18 overlapped ligands are listed.

Please see Supplementary Data 31 for the full list.

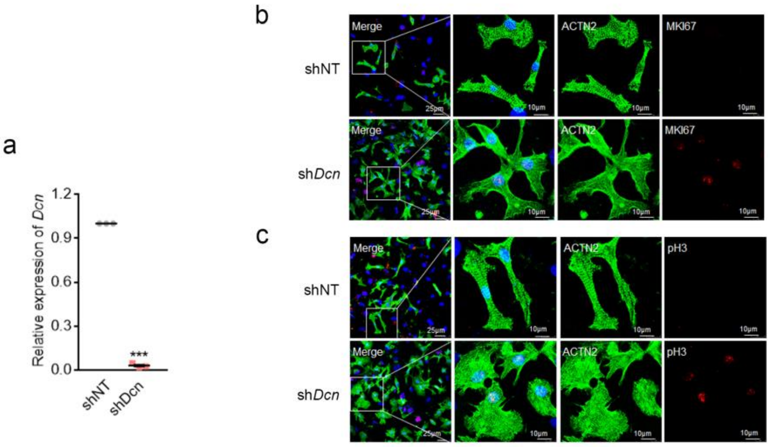

Supplementary Figure 5. Targeted inhibition of conserved pathways impairs maturation *in vitro*. (a) Real-time PCR to show knockdown efficiency of *Dcn* after lentivirus infection. Data are plotted as mean ± SEM from 3 independent experiments, ***$p < 0.001$, two-sided Student's *t*-test. **(b-c)** Immunofluorescent (IF) staining against ACTN2 and MKI67 (b) or pH3 (c) in imCMs-AF upon transduction with shNT and sh*Dcn*, respectively. DAPI was applied to stain nuclei. Scale bar = 25 or 10 μm. Source data are provided as a Source_Data_Extended_Data_Fig. 5.

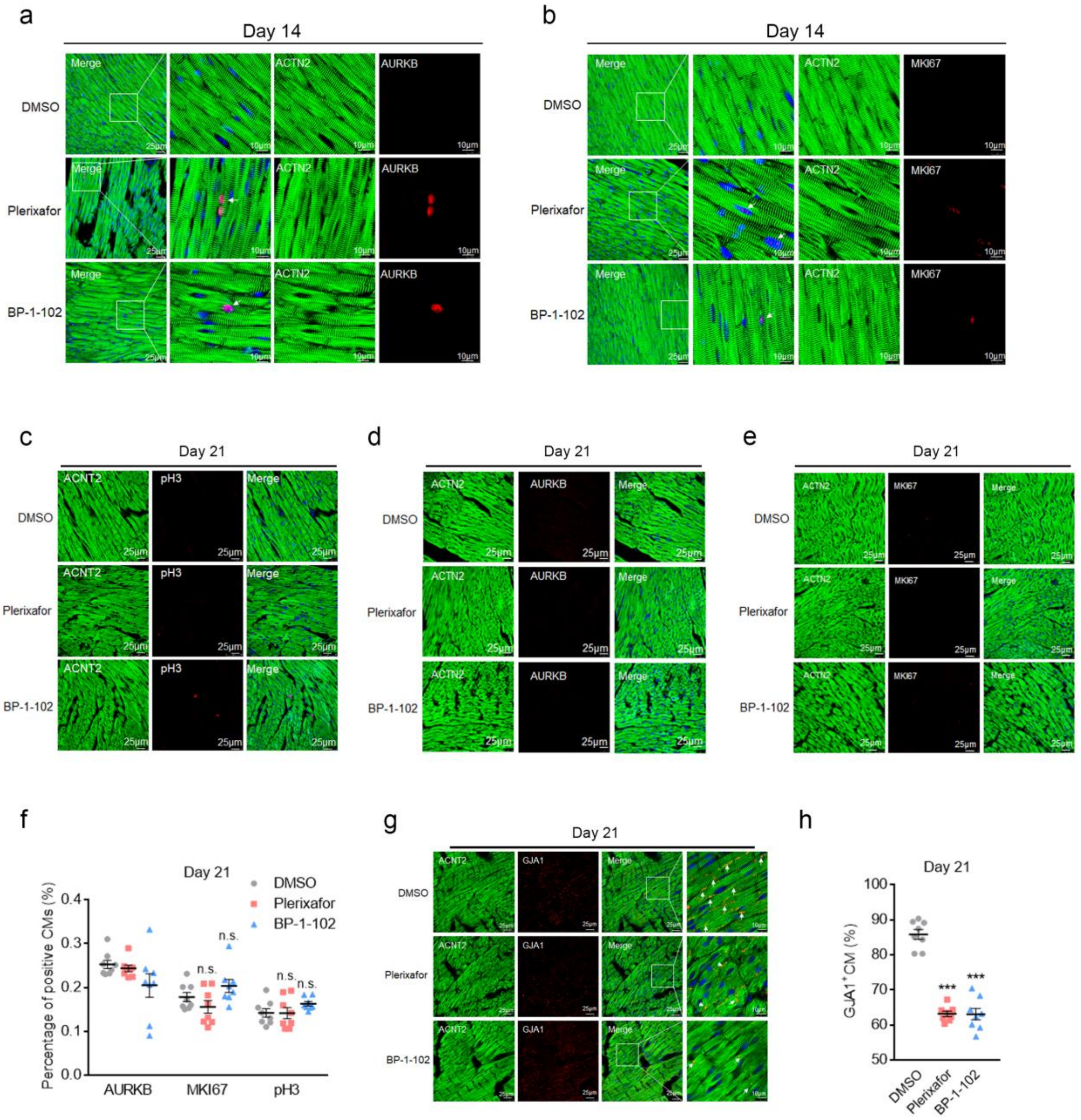

Supplementary Figure 6. Targeted inhibition of conserved pathways impairs maturation *in vivo*. (a-b) Immunofluorescent (IF) staining against ACTN2 and AURKB (a) or MKI67 (b) in heart sections from mice treated with DMSO, Plerixafor or BP-1-102 for 14 days, respectively. White arrows indicate co-localized cells. Scale bar = 10 or 25 µm. **(c-e)** Immunofluorescent (IF) staining against ACTN2 and pH3 (c), AURKB (d) or MKI67 (e) in heart sections from mice treated with DMSO, Plerixafor or BP-1-102 for 21 days, respectively. Scale bar = 25 µm. **(f)** Quantification of ACTN2^{+}pH3^{+}, ACTN2^{+}AURKB^{+}, and ACTN2^{+}MKI67^{+} cells. Data are plotted as mean ± SEM, n = 8 biologically independent mice,

n.s., not significant, two-sided Student's *t*-test. **(g)** Immunofluorescent staining of ACTN2 and GJA1 in heart sections from mice treated with DMSO, Plerixafor or BP-1-102 for 21 days, respectively. White arrows indicate co-localization. Scale bar = 10 or 25 μm. **(h)** Quantification of $ACTN2^{+}GJA1^{+}$ cells in (g). Data are plotted as mean ± SEM, n = 8 biologically independent mice, ****p* < 0.001, two-sided Student's *t*-test. Source data are provided as a Source_Data_Extended_Data_Fig. 6.

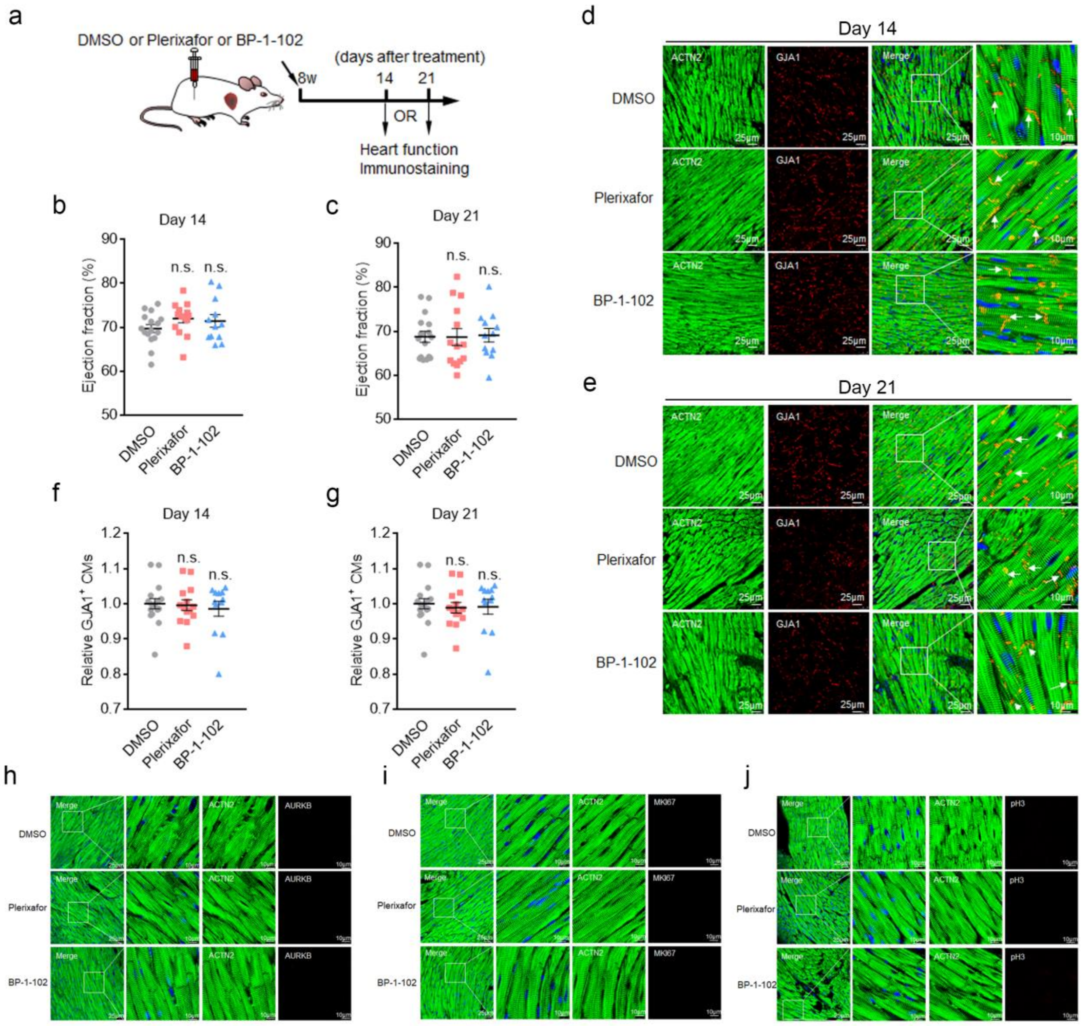

Supplementary Figure 7. Pathway inhibition is specific to immature CMs. (a) Workflow of animal experiments. Adult (P56) mice were treated with DMSO, Plerixafor or BP-1-102 for 14 (Day 14) or 21 days (Day 21), respectively. Cardiac function was evaluated at P14 or P21, respectively. **(b-c)** Ejection fraction of hearts at day 14 (b) or day 21 (c) was measured by echocardiography. Data are plotted as mean ± SEM, n = 16, 14, 12 biologically independent mice in DMSO, Plerixafor and BP-1-102 group, respectively, n.s., not significant, two-sided Student's *t*-test. **(d-e)** Immunofluorescent staining of ACTN2 and GJA1 in heart sections from mice treated with DMSO, Plerixafor or BP-1-102 for 14 (d) or 21 (e) days, respectively. White arrows indicate co-localized cells. Scale bar = 10 or 25 μm. **(f-g)**

Quantification of ACTN2$^+$GJA1$^+$ cells (d, e). Data are plotted as mean ± SEM, n = 16, 14, 12 biologically independent mice in DMSO, Plerixafor and BP-1-102 group, respectively, n.s., not significant, two-sided Student's *t*-test. **(h-j)** Immunofluorescent staining against ACTN2 and AURKB (h), MKI67 (i), or pH3 (j) in heart sections from mice treated with DMSO, Plerixafor or BP-1-102 for 14 days, respectively. Scale bar = 10 or 25 μm. Source data are provided as a Source_Data_Extended_Data_Fig. 7.

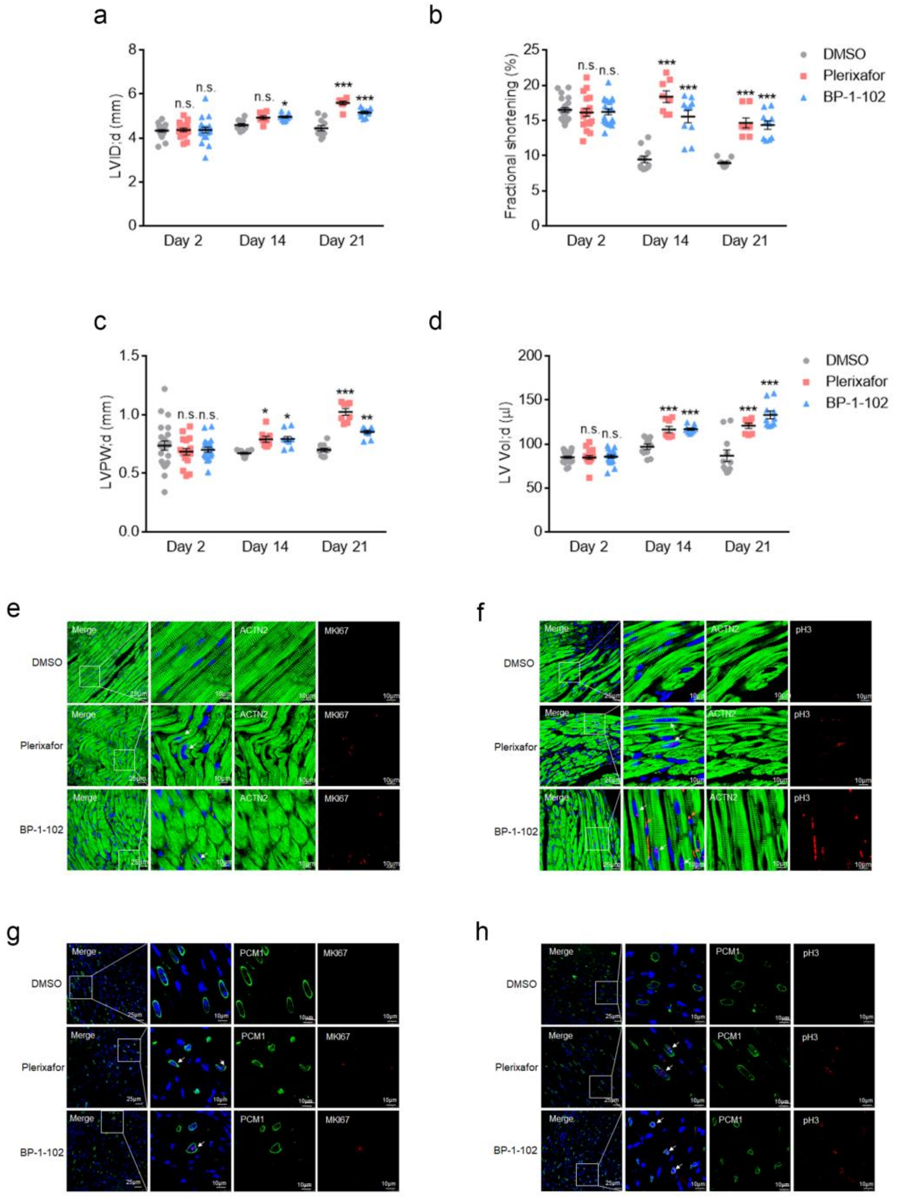

Supplementary Figure 8. Pathway inhibition reverses CM maturation upon injury. (a-d) Quantification of left ventricular end diastolic internal diameter (LVID;d, a), fractional shortening (b), left ventricular end diastolic posterior wall thickness (LVPW;d, c), and left ventricular end diastolic volume (LV Vol;d, d) based on echocardiogram. Data are plotted as mean ± SEM, n = 23, 18, 20 biologically independent mice in DMSO, Plerixafor and BP-1-102 group at Day 2, respectively, n = 11, 8, 10 biologically independent mice in DMSO, Plerixafor and BP-1-102 group at Day 14 and Day 21, respectively, *$p < 0.05$, **$p < 0.01$,

***$p < 0.001$, n.s., not significant, two-way ANOVA. **(e-f)** Immunofluorescent staining against ACTN2 and MKI67 (e) or pH3 (f) in border zones of heart sections from MI mice treated with DMSO, Plerixafor or BP-1-102 for 14, respectively. White arrows indicate co-localized cells. Scale bar = 10 or 25 μm. **(g-h)** Immunofluorescent staining against PCM1 and MKI67 (g) or pH3 (h) in the border zones of infarcted heart sections. White arrows indicate co-localized cells. Scale bar = 10 or 25 μm. Source data are provided as a Source_Data_Extended_Data_Fig. 8.

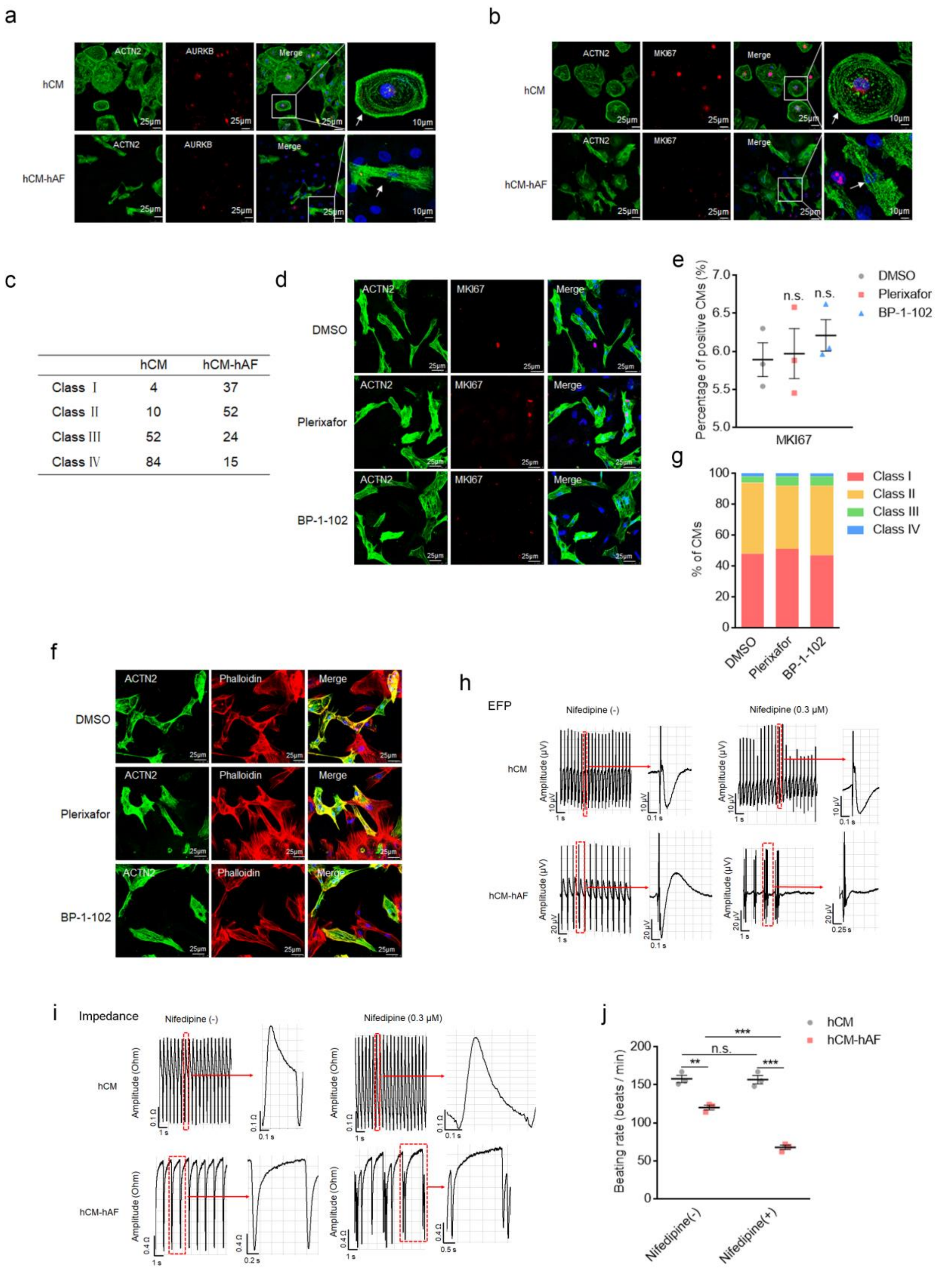

Supplementary Figure 9. Conserved role of FBs in human cell model. (a-b) Immunofluorescent (IF) staining against ACTN2 and AURKB (a) or MKI67 (b) in hESC-CMs 7 days after co-culture with or without hAFs, respectively. White arrows indicate co-localized cells. Scale bar = 10 or 25 μm. **(c)** Number of CMs in different morphology

grades in the presence or absence of hAFs. **(d)** Immunostaining against ACTN2 and MKI67 in hESC-CMs after co-culture with hAFs in the presence of DMSO, Plerixafor or BP-1-102, respectively. **(e)** Percentage of $ACTN2^{+}MKI67^{+}$ CMs in (d). Data are plotted as mean ± SEM from 3 independent experiments, ***p < 0.001, two-sided Student's *t*-test. **(f)** Immunostaining against ACTN2 and Phalloidin in hESC-CMs after co-culture with hAFs in the presence of DMSO, Plerixafor or BP-1-102, respectively. **(g)** Percentage of CMs in different morphology grades in (f). **(h-i)** Representative tracings to display cell extracellular field potential (EFP) (h) and impedance (i) in the presence or absence of nifedipine (0.3 μM) treatment. **(j)** Quantification of beating rates. Data are plotted as mean ± SEM from 3 independent experiments, **p < 0.01, ***p < 0.001, n.s., not significant, two-sided Student's *t*-test. Source data are provided as a Source_Data_Extended_Data_Fig. 9.
