# Supplementary material for: Single-cell analysis of murine fibroblasts identifies neonatal to adult switching that regulates cardiomyocyte maturation
Source: Nat Commun. 2020 May 22;11:2585. doi: 10.1038/s41467-020-16204-w (PMC7244751; doi:10.1038/s41467-020-16204-w)
Supplement: Supplementary file 2 — Reporting Summary [file 41467_2020_16204_MOESM2_ESM.pdf]

nature research

Corresponding author(s): Li Wang

Last updated by author(s): Apr 3, 2020

Reporting Summary

Nature Research wishes to improve the reproducibility of the work that we publish. This form provides structure for consistency and transparency in reporting. For further information on Nature Research policies, see Authors & Referees and the Editorial Policy Checklist .

Statistics

For all statistical analyses, confirm that the following items are present in the figure legend, table legend, main text, or Methods section.

n/a	Confirmed	
☐	☒	The exact sample size (n) for each experimental group/condition, given as a discrete number and unit of measurement
☐	☒	A statement on whether measurements were taken from distinct samples or whether the same sample was measured repeatedly
☐	☒	The statistical test(s) used AND whether they are one- or two-sided<br>*Only common tests should be described solely by name; describe more complex techniques in the Methods section.*
☒	☐	A description of all covariates tested
☒	☐	A description of any assumptions or corrections, such as tests of normality and adjustment for multiple comparisons
☒	☐	A full description of the statistical parameters including central tendency (e.g. means) or other basic estimates (e.g. regression coefficient) AND variation (e.g. standard deviation) or associated estimates of uncertainty (e.g. confidence intervals)
☐	☒	For null hypothesis testing, the test statistic (e.g. F, t, r) with confidence intervals, effect sizes, degrees of freedom and P value noted<br>*Give P values as exact values whenever suitable.*
☒	☐	For Bayesian analysis, information on the choice of priors and Markov chain Monte Carlo settings
☒	☐	For hierarchical and complex designs, identification of the appropriate level for tests and full reporting of outcomes
☒	☐	Estimates of effect sizes (e.g. Cohen's d, Pearson's r), indicating how they were calculated

Our web collection on statistics for biologists contains articles on many of the points above.

Software and code

Policy information about availability of computer code

Data collection	Single-cell sequencing libraries were generated using the iCell8 platform (Takara), and were sequenced by Illumina NextSeq 500 and basecalled by bcl2fastq (2.18.0.12).
Data analysis	For single cell data analysis, Wafergen pipeline scripts, cutadapt (1.8.1), bowtie2 (2.2.4), fastq_screen (0.5.1), STAR (2.5.2b), subread (1.4.6-p1 command line), kg (0.10.0), R packages: Seurat (2.1.0), scater (1.10.1), SingleCellExperiment (1.4.1), Scran (1.8.4), monocle (2.6.0), network (1.13.0.1), sna (2.4) ,GGally (1.4.0) and circlize (0.4.2).

For manuscripts utilizing custom algorithms or software that are central to the research but not yet described in published literature, software must be made available to editors/reviewers. We strongly encourage code deposition in a community repository (e.g. GitHub). See the Nature Research guidelines for submitting code & software for further information.

Data

Policy information about availability of data

All manuscripts must include a data availability statement. This statement should provide the following information, where applicable:

- Accession codes, unique identifiers, or web links for publicly available datasets
- A list of figures that have associated raw data
- A description of any restrictions on data availability

The authors declare that all data supporting the findings of this study are available within the article and its supplementary information files or from the corresponding author upon reasonable request. Raw sequencing data generated in this study are publicly available through the GEO database (GSE123547). Single-cell RNA-Seq data of mouse cardiomyocytes in postnatal P1 to P14 (GSE122706) were generated in a completely separate study by our group using the same single-cell platform as in this study, and all publicly available. Corresponding codes can be accessed from GitHub using the following link https://github.com/LiWangLab/Cardiomyocyte_Maturation. The source data underlying Figs 3d-e, 4c, h-m, o, q, r, t, v, 7b, i, k, 8b, d, f, 9c, and 9i-n and Supplementary Figs 3e-i, 5a, 6f, h, 7b-c, f-g, 8a-d, 9e and 9j are provided as a Source Data file.

Field-specific reporting

Please select the one below that is the best fit for your research. If you are not sure, read the appropriate sections before making your selection.

- [x] Life sciences
- [ ] Behavioural & social sciences
- [ ] Ecological, evolutionary & environmental sciences

For a reference copy of the document with all sections, see nature.com/documents/nr-reporting-summary-flat.pdf

Life sciences study design

All studies must disclose on these points even when the disclosure is negative.

Sample size	No sample size calculation was performed. We chose the corresponding sample size based on the literature which was sufficient to analyze significance between groups.
Data exclusions	Data exclusions were only performed in single-cell RNA-Seq analysis. We applied the standard filtering step to exclude cells with low quality based on the criteria from the literature.
Replication	All data were generated from 3 independent experiments unless otherwise specified.
Randomization	Both C57BL/6 mouse and cultured cells were allocated into experimental groups in random for single-cell RNA-seq, bulk RNA-seq , and animal experiments.
Blinding	The investigators were blinded to group allocate during data collection and analyse.

Reporting for specific materials, systems and methods

We require information from authors about some types of materials, experimental systems and methods used in many studies. Here, indicate whether each material, system or method listed is relevant to your study. If you are not sure if a list item applies to your research, read the appropriate section before selecting a response.

Materials & experimental systems

n/a	Involved in the study	
[ ]	[x]	Antibodies
[ ]	[x]	Eukaryotic cell lines
[x]	[ ]	Palaeontology
[ ]	[x]	Animals and other organisms
[ ]	[x]	Human research participants
[x]	[ ]	Clinical data

Methods

n/a	Involved in the study	
[x]	[ ]	ChIP-seq
[ ]	[x]	Flow cytometry
[x]	[ ]	MRI-based neuroimaging

Antibodies

Antibodies used	Immunostaining with anti-ACTN2 (1:200, ab9465, Abcam, USA), anti-TNNT2 (1:100, ab8295, Abcam, USA), and anti-PCM1 (G-6) (1:50, sc-398365, Santa Cruz, USA) were used as cardiomyocyte markers. To examine cardiac proliferation, the following antibodies were used: monoclonal anti-AURKB antibody (1:100, ab2254, Abcam), anti-MKI67 antibody (1:100, ab15580, Abcam), anti-phospho-histone (pH3) antibody (1:100, 53348, CST). To examine cardiomyocyte maturation, the following antibodies were used: anti-GJA1 antibody (1:100, 15386-1-AP, proteintech), EGR1 antibody (1:100, 55117-1-AP, proteintech), FABP4 antibody (1:100, ab92501, Abcam). To examine fibroblast identity, the following antibodies were used: anti-VIM antibody (ab193555, Abcam), anti-VIM antibody (ab8978, Abcam). The TRITC-Phalloidin reagent (1:300, Thermo Fisher) was used to visualize F-actin for morphology assessment. The secondary antibodies used in this study were fluorescein (FITC488)-conjugated goat anti-mouse IgG (H+L) (1:400, ZA-0511, ZSGB-BIO, China) and Alex Fluor 594-conjugated goat anti-rabbit IgG (H+L) (1:400, ZA-0512, ZSGB-BIO, China). FITC488-conjugated goat anti-rabbit IgG (H+L) (1:400, ZA-0513, ZSGB-BIO, China), Alex Fluor 594-conjugated goat anti-mouse IgG (H+L) (1:400, ZA-0514, ZSGB-BIO, China), and FITC488-conjugated goat anti-rat IgG (H+L) (1:400, ZA-0515, ZSGB-BIO, China). Cell nuclei were counterstained with 0.1% 4', 6-diamidino-2-phenylindole (DAPI, ZSGB-BIO, China).
Validation	All of the antibodies were validated by the manufacturer, related citations or literature.

Eukaryotic cell lines

Policy information about cell lines

Cell line source(s)	H1 was purchased and authenticated from ATCC.

Authentication	The use of H1 cell line were directly authenticated by ATCC when purchasing it.
Mycoplasma contamination	All cell lines tested negative for mycoplasma contamination.
Commonly misidentified lines (See ICLAC register)	No commonly misidentified cell lines were used in the study.

Animals and other organisms

Policy information about studies involving animals; ARRIVE guidelines recommended for reporting animal research

Laboratory animals	Eight- to10-week-old male C57BL/6 mice were used in this study unless otherwise specified. All mice were housed in standard SPF condition with temperatures of 65-75°F (~18-23°C) and with 40-60% humidity.
Wild animals	The study did not involve wild animals.
Field-collected samples	The study did not involve field-collected samples.
Ethics oversight	All animal protocols were approved by the Institutional Animal Care and Use Committee, Experimental Animal Center, Fuwai Hospital, National Center for Cardiovascular Diseases, China.

Note that full information on the approval of the study protocol must also be provided in the manuscript.

Human research participants

Policy information about studies involving human research participants

Population characteristics	Adult human hearts tissues were resected from healthy donors with failed matching in heart transplantation from the clinic. The donor with age ranging from 20-45 years.
Recruitment	Adult human hearts tissues were resected from healthy donors with failed matching in heart transplantation from the clinic. No bias were introduced in this study.
Ethics oversight	For the use of human tissue, informed consent was obtained from subjects in the clinic for use of the human heart tissue in research.

Note that full information on the approval of the study protocol must also be provided in the manuscript.

Flow Cytometry

Plots

Confirm that:

- [x] The axis labels state the marker and fluorochrome used (e.g. CD4-FITC).
- [x] The axis scales are clearly visible. Include numbers along axes only for bottom left plot of group (a 'group' is an analysis of identical markers).
- [x] All plots are contour plots with outliers or pseudocolor plots.
- [x] A numerical value for number of cells or percentage (with statistics) is provided.

Methodology

Sample preparation	Adult cardiac fibroblasts (passages 3-6) and neonatal imCMs (P1), from C57BL/6J mice were stained by CellTrackerTM Green CMFDA (Thermo, C2925) at 37℃ for 20 min, respectively, whereas imCMs were stained with CellTrackerTM Res CMTPX (Invitrogen, C34552) at 37℃ for 20 min. Flow cytometry was performed to separate them after co-culture.
Instrument	BD FACS CantoTM II flow cytometry system (BD Biosciences)
Software	DIVA
Cell population abundance	100,000~300,000
Gating strategy	CellTrackerTM Green CMFDA: 488nm; CellTrackerTM Res CMTPX: 594nm

- [x] Tick this box to confirm that a figure exemplifying the gating strategy is provided in the Supplementary Information.
