# Supplementary material for: Single-cell analysis of murine fibroblasts identifies neonatal to adult switching that regulates cardiomyocyte maturation
Source: Nat Commun. 2020 May 22;11:2585. doi: 10.1038/s41467-020-16204-w (PMC7244751; doi:10.1038/s41467-020-16204-w)
Supplement: Supplementary file 3 — Description of Additional Supplementary Files [file 41467_2020_16204_MOESM3_ESM.pdf]

File Name	Legend
Supplementary Data 1	Enriched pathways in State 1 in CM pseudotime. Functional analysis was performed with enrichGO in clusterProfiler. $P < 0.05$ was considered significant enrichment.
Supplementary Data 2	Enriched pathways in State 2 in CM pseudotime. Functional analysis was performed with enrichGO in clusterProfiler. $P < 0.05$ was considered significant enrichment.
Supplementary Data 3	Enriched pathways in State 7 in CM pseudotime. Functional analysis was performed with enrichGO in clusterProfiler. $P < 0.05$ was considered significant enrichment.
Supplementary Data 4	Enriched pathways in State 6 in CM pseudotime. Functional analysis was performed with enrichGO in clusterProfiler. $P < 0.05$ was considered significant enrichment.
Supplementary Data 5	Enriched pathways in State 8 in CM pseudotime. Functional analysis was performed with enrichGO in clusterProfiler. $P < 0.05$ was considered significant enrichment.
Supplementary Data 6	Enriched pathways in State 9 in CM pseudotime. Functional analysis was performed with enrichGO in clusterProfiler. $P < 0.05$ was considered significant enrichment.
Supplementary Data 7	Top 1000 DEGs along the pseudotime trajectory *in vivo*. *P* values and *q* values were calculated from the likelihood ratio tests in differentialGeneTest in Monocle (v2.6.0). DEGs were ranked by ascending *q* values, and the top 1000 were selected.
Supplementary Data 8	Full list of DEGs in EC cell clusters *in vivo*. Differentially expressed genes were identified using a Wilcoxon Rank Sum test with FindAllMarkers in Seurat (v2.1.0).
Supplementary Data 9	Full list of DEGs in FB cell clusters *in vivo.* Differentially expressed genes were identified using a Wilcoxon Rank Sum test with FindAllMarkers in Seurat (v2.1.0).
Supplementary Data 10	Full list of DEGs in MP cell clusters *in vivo.* Differentially expressed genes were identified using a Wilcoxon Rank Sum test with FindAllMarkers in Seurat (v2.1.0).
Supplementary Data 11	Full list of DEGs in SMC cell clusters *in vivo.* Differentially expressed genes were identified using a Wilcoxon Rank Sum test with FindAllMarkers in Seurat (v2.1.0).
Supplementary Data 12	Cell distribution in P1 and P56.
Supplementary Data 13	Enriched pathway *in vivo* in P1 and P56.
Supplementary Data 14	Secretory protein list of representative cell clusters *in vivo*. Differentially expressed genes were identified using a Likelihood-ratio test with FindAllMarkers in Seurat (v2.1.0).
Supplementary Data 15	Interaction between nonCM to CM clusters *in vivo* in P1 and P56.
Supplementary Data 16	DEGs in P1 vs. P56 FBs. Differentially expressed genes were identified using a Likelihood-ratio test with FindMarkers in Seurat (v2.1.0).

Supplementary Data 17	Top 1000 DEGs along the FB pseudotime trajectory *in vivo*. *P* values and *q* values were calculated from the likelihood ratio tests in differentialGeneTest in Monocle (v2.6.0). DEGs were ranked by ascending *q* values, and the top 1000 were selected.
Supplementary Data 18	Enriched pathways in cluster 3 along FB pseudotime.
Supplementary Data 19	Enriched pathways in cluster 1 along FB pseudotime.
Supplementary Data 20	Enriched pathways in cluster 4 along FB pseudotime.
Supplementary Data 21	Signaling hotspots in P1.
Supplementary Data 22	Signaling hotspots in P56.
Supplementary Data 23	Enriched pathways of sigHotspot in condition 1_P1. Functional analysis was performed with enrichKEGG in clusterProfiler. $P < 0.05$ was considered significant enrichment.
Supplementary Data 24	Enriched pathways of sigHotspot in condition 2_P56. Functional analysis was performed with enrichKEGG in clusterProfiler. $P < 0.05$ was considered significant enrichment.
Supplementary Data 25	Full list of up- and down-regulated genes in RNA-Seq. Differentially expressed genes were identified using linear model with empirical Bayes moderated t-statistics in Limma.
Supplementary Data 26	logTPM of upregulated and downregulated genes in RNASeq.
Supplementary Data 27	DEGs along the pseudotime trajectory *in vitro.*
Supplementary Data 28	Enriched pathway *in vitro.*
Supplementary Data 29	Secretory protein list of co-cultured FB *in vitro*. Differentially expressed genes were identified using a Likelihood-ratio test with FindAllMarkers in Seurat (v2.1.0).
Supplementary Data 30	Unique pathways *in vivo* and *in vitro.*
Supplementary Data 31	Unique ligands *in vivo* and *in vitro.*
Supplementary Data 32	Mitochondrial Genes.
Supplementary Data 33	Full list of housekeeping genes.
